# Fe_3_O_4_ Magnetic Nanoparticles and Static Magnetic Field Stimulated BMSC-Derived Exosomes Promoted Osteogenesis and Alleviated Oxidative Stress in Irradiated BMSCs Through miR-429/NOG Pathway

**DOI:** 10.3390/bioengineering13040402

**Published:** 2026-03-30

**Authors:** Ting Mou, Chong Huang, Zhiyue Zhang, Heng Li, Lu Zhao, Yuxin Bing, Dandan Wang, Lei Tian, Chunlin Zong

**Affiliations:** 1 School of Stomatology, Jiamusi University, Jiamusi 154007, China; mtmuting@126.com (T.M.);; 2 State Key Laboratory of Oral & Maxillofacial Reconstruction and Regeneration, National Clinical Research Center for Oral Diseases, Shaanxi Clinical Research Center for Oral Diseases, Department of Oral and Maxillofacial Surgery, School of Stomatology, The Fourth Military Medical University, Xi’an 710032, China; huangchongson@163.com (C.H.); zhangzy126830@163.com (Z.Z.);

**Keywords:** radiation-induced bone injury, magnetic nanoparticles, static magnetic field, exosomes, miR-429, NOG, bone regeneration, ROS

## Abstract

Radiation-induced bone injury, characterized by oxidative stress damage and impaired osteogenesis, lacks effective treatments. Exosome-based therapies have recently emerged as a safe and effective modality for radiation damage, and their functional capacity can be further potentiated through tailored preconditioning strategies—such as nanoparticle induction or physical stimulation. This study developed a novel exosome-based therapy by preconditioning bone marrow mesenchymal stem cells (BMSCs) with Iron oxide (Fe_3_O_4_) magnetic nanoparticles (MNPs, 50 µg/mL) and a static magnetic field (SMF, 100 mT). Exosomes derived from these preconditioned cells (BMSC-Fe_3_O_4_-SMF-Exos) exhibited enhanced yield and dual functionality. In irradiated BMSCs, BMSC-Fe_3_O_4_-SMF-Exos significantly promoted osteogenic differentiation, restoring alkaline phosphatase activity, mineralization, and expression of RUNX2, OCN, and COL1A1. They concurrently alleviated oxidative stress by scavenging reactive oxygen species, reducing malondialdehyde, and boosting superoxide dismutase activity. Mechanistically, miR-429 was found to be highly enriched in BMSC-Fe_3_O_4_-SMF-Exos, which directly targeted Noggin (NOG). Our functional validation experiments also confirmed that overexpression of miR-429 could inhibit NOG, alleviate oxidative stress and rescue the osteogenic differentiation of irradiated BMSCs. In conclusion, exosomes derived from preconditioning BMSCs with Fe_3_O_4_ MNPs and SMF mitigate radiation-induced damage via the miR-429/NOG pathway, presenting a promising cell-free strategy for bone regeneration.

## 1. Introduction

Radiotherapy, as a cornerstone of cancer treatment, inevitably causes damage to surrounding healthy tissues [1]. Among the various radiation-induced injuries, radiation-induced bone injury is a particularly serious and often irreversible sequela [2,3]. Clinically, patients typically experience persistent local bone pain and an increased susceptibility to pathological fractures. When the condition progresses to an advanced stage, it can manifest as exposed necrotic bone, chronic infection, and even lead to dysfunction [4,5]. These complications severely impair patients’ physical function, nutritional status, and psychosocial well-being, causing a significant decline in their quality of life. Despite substantial advancements in radiotherapy techniques, addressing radiation-induced bone injury from both preventive and therapeutic standpoints has long persisted as a critical, unresolved challenge in routine clinical practice due to its complex pathogenesis and the lack of highly effective therapeutic approaches [6].

The pathogenesis is initiated by a persistent oxidative stress outburst within the irradiated bone marrow niche [7], driven by excessive reactive oxygen and nitrogen species (ROS/RNS) [8]. This cascade inflicts direct macromolecular damage and impairs the function and viability of BMSCs—the pivotal progenitors for bone regeneration [9]. Concurrent microvascular rarefaction and endothelial apoptosis further exacerbate the damage, creating a hypoxic and nutrient-deprived microenvironment [10]. This dual assault on cellular and structural integrity disrupts bone homeostasis, promoting sustained osteoclast activity while suppressing osteogenesis, which culminates in osteoporosis, impaired healing, and osteoradionecrosis [11,12].

Recently, BMSC-derived exosomes have emerged as a promising cell-free therapy [13]. They retained the osteogenic and immunomodulatory functions of BMSCs, targeted injured tissues, and avoided immune rejection [14,15]. These nanosized vesicles can mitigate oxidative stress and promote tissue repair by delivering bioactive cargo. Preclinical studies in radiation-induced bone damage models supported their therapeutic potential [16,17,18,19]. However, clinical translation of natural exosomes is hindered by poor targeting, low drug loading, rapid clearance, and batch heterogeneity [20]. To overcome these limitations, various modified strategies, such as preconditioning parent cells, were actively explored to enhance the potency and specificity of exosomes [21]. For instance, BMSCs were pretreated with electrical stimulation, and the derived exosomes were able to activate osteogenesis-related core signaling cascades, exemplified by the well-documented canonical PI3K/Akt and MAPK signaling axes in recipient cells [22], thereby providing an innovative combined therapeutic strategy for bone regeneration.

Extensive research has confirmed the ability of Fe_3_O_4_ MNPs to boost the proliferative ability and osteogenic differentiation potential of stem cells—especially mesenchymal stem cells—as well as osteoblasts [23,24]. Among various MNPs, iron oxide (Fe_3_O_4_) MNPs stand out as one of the most extensively studied materials, owing to their inherent biocompatibility, low systemic toxicity, natural presence in biological systems, and favorable in vivo tolerability [25]. Accumulating evidence underscores the ability of Fe_3_O_4_ magnetic nanoparticles (MNPs) to enhance the proliferative activity and osteogenic differentiation potential of stem cells, especially mesenchymal stem cells, as well as osteoblasts. In particular, multiple in vitro and in vivo experimental data have shown that Fe_3_O_4_ MNPs effectively enhance the osteogenic differentiation of BMSCs [26]. Static magnetic fields (SMFs) have been shown to influence various cellular processes, including proliferation, differentiation, and paracrine signaling, through mechanisms such as mechanotransduction, modulation of ion channel activity, and reorganization of the cytoskeleton [27,28]. When combined with Fe_3_O_4_ magnetic nanoparticles (MNPs), SMF can exert mechanical forces on the nanoparticles internalized by cells, potentially amplifying these biological effects and promoting the enrichment of specific therapeutic miRNAs in secreted exosomes [29,30]. More intriguingly, when in conjunction with a static magnetic field (SMF), Fe_3_O_4_ MNPs can further modulate the paracrine function of BMSCs by regulating the content of exosomes they secrete. These magnetically conditioned exosomes (e.g., BMSC-Fe_3_O_4_-SMF-Exos and mag-BMSC-Exos) have been verified to exert superior biological effects, in which exosome-encapsulated miR-1260a facilitated osteogenic and angiogenic processes by directly targeting HDAC7 and COL4A2, while the upregulation of miR-21-5p and subsequent SPRY2 inhibition had enhanced angiogenesis and fibroblast activity, thereby accelerating cutaneous wound repair [29,30]. Such findings implied the synergistic potential of Fe_3_O_4_ MNPs and SMF in optimizing exosome-mediated therapeutic effects for bone regeneration and wound repair. Among the miRNAs implicated in bone metabolism and oxidative stress regulation, miR-429 has been reported to protect osteoblastic cells from injury by activating the AMPK pathway and reducing reactive oxygen species [31]. However, its role in radiation-induced bone injury and its potential as a cargo of magnetically conditioned exosomes have not been explored. Based on this discovery, we propose to innovatively apply it to a specific and challenging clinical scenario, the treatment of radiation-induced bone injuries.

While previous studies have extensively investigated the direct application of nanoparticles as scaffolds or delivery systems in bone tissue engineering [4,32], and have demonstrated that bone-derived nanoparticles (BNPs) can directly promote osteogenic differentiation via Notch signaling [33,34], the potential of using nanoparticles as cellular priming agents to generate functionally enhanced exosomes—particularly for the treatment of radiation-induced bone injury—remains largely unexplored. Furthermore, although Fe_3_O_4_ MNPs combined with SMF have been shown to enrich therapeutic miRNAs in exosomes for wound healing [29,30], their application in bone regeneration, and specifically in mitigating radiation-induced damage, has not been reported. This study addresses these gaps by demonstrating for the first time that Fe_3_O_4_/SMF-preconditioned BMSC-derived exosomes promote osteogenesis and alleviate oxidative stress in irradiated BMSCs via the miR-429/NOG pathway, offering a novel cell-free strategy for radiation-induced bone injury.

This study systematically evaluated the curative efficacy of BMSC-Fe_3_O_4_-SMF-Exos on radiation-damaged BMSCs. By integrating exosomal miRNA sequencing, transcriptome sequencing, and functional validation, the underlying mechanism was elucidated, thereby laying the groundwork for the development of advanced exosome-based regenerative therapies for radiation-induced bone damage.

## 2. Materials and Methods

### 2.1. Isolation and Culture of BMSCs

Bone marrow mesenchymal stem cells (BMSCs) were obtained from 2 to 3-week-old male Sprague-Dawley (SD) rats provided by the Laboratory Animal Center of Air Force Medical University. All animal experiments were reviewed and approved by the Ethics and Laboratory Animal Welfare Committee of the Fourth Military Medical University (Approval No.: 20250202). After euthanasia, BMSCs were isolated from the bilateral femurs and tibias of rats using the whole bone marrow adherent culture method [35]. Detailed procedures were as described below. Following euthanasia via pentobarbital sodium (MW: 248.26 g/mol, ≥99.0% purity, Sigma-Aldrich, St. Louis, MO, USA) overdose, the bilateral femurs and tibias were quickly separated, and the attached soft tissues were removed aseptically. Bone marrow was extracted by flushing with complete medium, and the collected cell suspension was then plated into culture flasks. Cells were cultured in α-Minimum Essential Medium (α-MEM; Gibco, Waltham, MA, USA) containing 10% heat-inactivated fetal bovine serum (FBS, pH 7.4; Beyotime Biotechnology, Shanghai, China), 100 U/mL penicillin, and 100 μg/mL streptomycin (Gibco, USA). All cells were kept in a humidified atmosphere of 5% CO_2_ at 37 °C, and the culture medium was refreshed every 72 h. Upon reaching 80–90% confluence, cells were detached using 0.25% trypsin-EDTA (Gibco, USA) and subjected to subsequent subculture. BMSCs at passages 3–5 (P3–P5) were used for all subsequent experiments. To confirm their stem cell properties, BMSCs were identified through immunophenotypic analysis.

### 2.2. Flow Cytometry Analysis

The immunophenotype of the cultured BMSCs was identified via flow cytometry. BMSCs at the 3rd passage were collected and subsequently resuspended in PBS. Cell suspensions were then treated with fluorochrome-conjugated antibodies specific for CD29 (1:50, Cat No. GTX43634, GeneTex), CD90 (1:100, Cat No. GTX76208, GeneTex), CD34 (1:100, Cat No. GTX02602, GeneTex), and CD45 (1:100, Cat No. GTX43587, GeneTex, San Antonio, TX, USA) for 30 min at room temperature in the dark. Isotype-matched antibodies were used under identical conditions as negative controls. After incubation and washing, the cells were analyzed using a flow cytometer (Beckman Coulter, USA) with a 488 nm excitation laser; 1 × 10^4^ valid cells were collected per sample, and data were analyzed using the CytExpert 2.4 software (Beckman Coulter, Brea, CA, USA).

### 2.3. Synthesis, Characterization of Fe_3_O_4_ MNPs and Setup of SMF

Fe_3_O_4_ MNPs were synthesized via a modified solvothermal method [36]. In brief, 40 mL ethylene glycol (EG, MW: 62.07 g/mol, ≥99.5% purity, Sigma-Aldrich, St. Louis, MO, USA) was used to dissolve 1.35 g of Iron(III) chloride hexahydrate (FeCl_3_·6H_2_O, MW: 270.30 g/mol, ≥99.0% purity, Sigma-Aldrich, St. Louis, MO, USA) initially. Thereafter, 1.0 g polyethylene glycol 4000 (PEG 4000, MW: 3800–4200 g/mol, ≥99.0% purity, Sigma-Aldrich, St. Louis, MO, USA) and 3.6 g sodium acetate trihydrate (NaAc·3H_2_O, MW: 136.08 g/mol, ≥99.0% purity, Sigma-Aldrich, St. Louis, MO, USA) were added to this solution, and the mixture was intensely agitated until it was completely dissolved. The transparent mixture was then placed into a 50 mL Teflon-lined stainless steel autoclave, with heating conducted at 200 °C for a 72 h period. After the system cooled to ambient temperature, the black precipitate formed was harvested, rinsed three times with anhydrous ethanol (MW: 46.07 g/mol, ≥99.7% purity, Sinopharm Chemical Reagent Co., Ltd., Shanghai, China) and deionized water, and dried under vacuum at 60 °C for subsequent use. A stock dispersion of Fe_3_O_4_ MNPs (200 µg/mL) was prepared by dispersing 10 mg of the powder in 50 mL of basal culture medium via vortexing and ultrasonication, followed by incubation at 37 °C for 24 h. This stock dispersion was then serially diluted with culture medium to obtain working concentrations of 25, 50, 100 and 200 µg/mL.

The synthesized MNPs were characterized for their morphology, composition, and crystal structure. Morphology and size were examined by transmission electron microscopy (TEM; FEI Tecnai G2 Spirit, Thermo Scientific, Waltham, MA, USA) operating at an acceleration voltage of 120 kV. Samples were prepared by dropping 10 µL of MNPs dispersion onto a 300-mesh carbon-coated copper grid, followed by natural drying at room temperature before detection. For particle size distribution analysis, the diameter of a total of 36 randomly selected Fe_3_O_4_ MNPs (from multiple representative TEM images) was measured using ImageJ software (v1.53, NIH, Bethesda, MD, USA). The particle size distribution was fitted with a Gaussian function, and the average particle size (D) and standard deviation (SD) were calculated and presented as D ± SD. The corresponding particle size distribution histogram is provided in the Supplementary Materials as Figure S2.

Scanning electron microscopy (SEM; ZEISS Sigma 300, ZEISS, Oberkochen, Germany) was performed at an acceleration voltage of 5 kV and a working distance of 8–10 mm. Samples were sputter-coated with a 10 nm gold layer prior to imaging to enhance conductivity. The elemental composition and surface chemical state were analyzed by energy-dispersive X-ray spectroscopy (EDS; OXFORD Xplore, Oxford Instruments plc, Abingdon, UK) coupled to the SEM system, operating at an acceleration voltage of 20 kV, with an acquisition time of 100 s per mapping area. The surface chemical state was analyzed by X-ray photoelectron spectroscopy (XPS; Thermo Scientific K-Alpha, Waltham, MA, USA) using a monochromatic Al Kα X-ray source (hν = 1486.6 eV) operating at 15 kV and 10 mA. Survey spectra were recorded with a pass energy of 100 eV and a step size of 1 eV, while high-resolution narrow spectra were acquired with a pass energy of 20 eV and a step size of 0.05 eV. All binding energies were calibrated using the adventitious C 1s peak at 284.8 eV. The crystal structure was determined by X-ray diffraction (XRD; Rigaku SmartLab SE, Rigaku Corporation, Tokyo, Japan) using Cu Kα radiation (λ = 0.15406 nm) operating at 40 kV and 40 mA. Diffraction patterns were collected in the 2θ range of 10° to 80°, with a scanning rate of 2°/min and a step size of 0.02°. The average crystallite size of Fe_3_O_4_ MNPs was calculated from the most intense (114) diffraction peak (2θ ≈ 35.5°) using the Scherrer equation: (D = 0.89\lambda/(\beta\cos\theta)) (FWHM ≈ 0.4°), yielding a crystallite size of ~18.4 nm. The hydrodynamic size distribution and zeta potential of the synthesized Fe_3_O_4_ MNPs were measured to evaluate their colloidal dispersion stability in aqueous solution. Tests were performed using a Zetasizer Nano ZS90 instrument (Malvern Instruments, Malvern, UK) at a constant temperature of 25 °C, with an equilibration time of 120 s before each measurement. Fe_3_O_4_ MNPs were dispersed in ultrapure water (pH 7.4) at a final concentration of 50 μg/mL (the optimal working concentration for subsequent BMSC co-culture experiments), followed by ultrasonic dispersion for 10 min to obtain a homogeneous suspension. Each sample was tested in 3 independent biological replicates, with 3 consecutive measurements per replicate, and the final data were presented as mean ± standard deviation (SD). The corresponding distribution diagrams are provided in the Supplementary Materials as Figure S1.

The SMF environment for cell culture was generated using neodymium-iron-boron (NdFeB) magnets (30 × 20 × 10 mm; N52 grade, Ganzhou Dingxi Magnetic Industry Co., Ltd., Ganzhou, China). The magnetic field intensities at the cell culture plane were set at 0, 50, 100, and 200 mT, as measured and confirmed by a gaussmeter (Model KT-101, AIRUIPU, Shenzhen, China). The desired magnetic field strength was obtained either by varying the gap between the magnets and the culture plate or by arranging the magnets in parallel stacks.

BMSCs were grown in standard culture medium alone or in medium containing the indicated different concentrations of Fe_3_O_4_ MNPs, with or without exposure to the various SMF intensities. The intracellular distribution of Fe_3_O_4_ MNPs within BMSCs was assessed by TEM (FEI Tecnai G2 Spirit, Thermo Scientific, Waltham, MA, USA) operating at an acceleration voltage of 80 kV. Cell samples were fixed, embedded, sectioned to 70 nm ultrathin slices, and stained with uranyl acetate and lead citrate before observation.

### 2.4. CCK-8 Assays

The optimal stimulation conditions (concentration of Fe_3_O_4_ MNPs and intensity of the SMF) were determined in a two-step process using a CCK-8 assay, followed by validation with live/dead cell staining. BMSCs were inoculated in 96-well plates with 5 × 10^3^ cells per well. After 12 h of adherence, cells were treated with culture medium containing Fe_3_O_4_ MNPs at concentrations of 0, 25, 50, 100 and 200 µg/mL without SMF exposure. According to the manufacturer’s protocol, cells were treated with 10% CCK-8 reagent (Beyotime, Shanghai, China) in fresh medium at 24, 48, and 72 h after incubation. The plates were incubated at 37 °C for 2 h, and the absorbance at 450 nm (OD450) was then measured using a microplate reader (Epoch, Missouri City, TX, USA). The optimal concentration—defined as the one yielding the highest cell viability—was chosen for follow-up experiments.

Using the optimal Fe_3_O_4_ MNPs concentration, BMSCs were co-cultured with the MNPs and simultaneously exposed to SMF at gradients of 0, 50, 100, and 200 mT. Cell viability was assessed using the same CCK-8 protocol at the aforementioned time points.

### 2.5. Live/Dead Cell Staining

To further verify biocompatibility under the optimized conditions, a Calcein-AM/PI Cell Viability/Cytotoxicity Assay Kit (Beyotime, China) was used. BMSCs were seeded in glass-bottom confocal dishes at densities of 1 × 10^5^ cells/dish (for optimal concentration verification) and 1 × 10^4^ cells/dish (for optimal SMF intensity verification) according to the manufacturer’s protocol and then subjected to the respective optimal treatments for 72 h. In accordance with the manufacturer’s protocol, cellular staining was performed using Calcein-AM (MW: 994.87 g/mol, ≥98.0% purity, Beyotime Biotechnology, Shanghai, China) (labeling live cells with green fluorescence) and propidium iodide (PI, MW: 668.40 g/mol, ≥95.0% purity, Beyotime Biotechnology, Shanghai, China) (PI, marking dead cells with red fluorescence). We then examined the stained cells and captured their images with a laser scanning confocal microscope (Olympus Corporation, Tokyo, Japan). For quantitative analysis, three random fields of view were collected per sample. The quantities of live (green) and dead (red) cells were measured with ImageJ software (NIH, Bethesda, MD, USA), and cell viability was computed as the percentage of live cells in the total cell population. We conducted all the experiments in three independent replicates, and the resulting data were reported as the mean ± standard deviation.

### 2.6. Exosome Isolation and Purification

When the BMSCs reached 70–80% confluency, we replaced the original culture medium with a standard growth medium added with 10% exosome-depleted FBS, and the cells were then further incubated for 48 h. We first collected the conditioned medium and subjected it to a series of differential centrifugations to isolate exosomes. Specifically, the culture medium was initially centrifuged at 300× *g* for 10 min, after which centrifugation was conducted at 2000× *g* for 20 min to eliminate cells and large cellular fragments. The subsequent supernatant was further centrifuged at 10,000× *g* for 30 min to precipitate larger vesicles. Following this, the supernatant was passed through a 0.22-μm filter (Merck-Millipore, Darmstadt, Germany) to eliminate particles exceeding 220 nm in size. Ultracentrifugation of the filtrate at 100,000× *g* for 70 min at 4 °C was performed to pellet the exosomes. The resulting pellet was resuspended in a suitable volume of PBS and underwent an additional ultracentrifugation at 110,000× *g* for 70 min at 4 °C for washing, so as to remove impurity proteins. All centrifugation steps were implemented at 4 °C. The final purified exosomal pellet was re-suspended in PBS and stored at −80 °C for subsequent experiments.

### 2.7. Exosome Characterization and Internalization

We characterized the particle size distribution and concentration of the isolated exosomes via nanoparticle tracking analysis (NTA) with a NanoSight NS500 system (Malvern Instruments, Malvern, UK) at a controlled temperature of 25 °C. The viscosity was set to match the aqueous buffer (PBS). Each sample was measured 3 consecutive times, with a 60 s acquisition duration per replicate, camera level set to 13, and detection threshold set to 5. Data were analyzed using the NTA 3.2 software (Malvern Instruments, Malvern, UK). For morphological observation, the exosomes were subjected to negative staining with uranyl acetate (MW: 424.15 g/mol, ≥98.0% purity, Ted Pella Inc., Redding, CA, USA) (2% *w*/*v*, 1 min staining) prior to TEM examination (FEI Tecnai G2 Spirit, Thermo Scientific, Waltham, MA, USA) operating at an acceleration voltage of 100 kV. Western blotting was performed to evaluate the expression of typical exosomal surface markers, including CD63, Flotillin-2 and TSG101. To explore how exosomes are internalized by BMSCs, we tagged the isolated exosomes with PKH26 red fluorescent dye (MW: 901.13 g/mol, ≥99.0% purity, Sigma-Aldrich, St. Louis, MO, USA) as recommended by the manufacturer’s instructions. Thereafter, BMSCs were incubated together with the PKH26-labeled exosomes at 37 °C for a 24 h period. After completing the co-incubation, the cells were rinsed thoroughly and fixed, and a confocal microscope (Nikon, Tokyo, Japan) was used to visualize the internalized exosomes and detect the corresponding PKH26 fluorescence.

### 2.8. Osteogenic Differentiation

Cells were randomly divided into five experimental groups according to the irradiation treatment and exosome intervention strategy. BMSCs in the irradiation groups were exposed to 6 Gy ionizing radiation using a Sharp-100pro X-ray irradiator.: (1) Control group: BMSCs without irradiation and treated with an equal volume of PBS instead of exosomes; (2) IR group: BMSCs with 6 Gy irradiation and treated with PBS; (3) BMSC-Exos group: BMSCs with 6 Gy irradiation and treated with 100 μg/mL BMSC-derived exosomes; (4) BMSC-Fe_3_O_4_-Exos group: BMSCs with 6 Gy irradiation and treated with 100 μg/mL exosomes from Fe_3_O_4_ MNPs-preconditioned BMSCs; (5) BMSC-Fe_3_O_4_-SMF-Exos group: BMSCs with 6 Gy irradiation and treated with 100 μg/mL exosomes from Fe_3_O_4_ MNPs and SMF-co-preconditioned BMSCs.

After BMSCs were co-incubated with exosomes or transfected with miRNA mimics/inhibitors for 24 h, we initiated osteogenic differentiation induction in the cells. More specifically, the initial culture medium was replaced with osteogenic induction medium. This medium was prepared on the basis of basal medium and supplemented with exosome-depleted FBS, penicillin-streptomycin, dexamethasone (MW: 392.46 g/mol, ≥99.0% purity, Sigma-Aldrich, St. Louis, MO, USA), ascorbic acid (MW: 176.12 g/mol, ≥99.0% purity, Sigma-Aldrich, St. Louis, MO, USA), and β-glycerophosphate (β-glycerophosphate, MW: 216.04 g/mol, ≥99.0% purity, Sigma-Aldrich, St. Louis, MO, USA). According to the assigned experimental groups, 200 μL of PBS or various exosome preparations (100 μg/mL for BMSC-Exos, BMSC-Fe_3_O_4_-Exos and BMSC-Fe_3_O_4_-SMF-Exos) was supplemented into the induction medium in line with the aforementioned experimental protocol. The osteogenic induction medium was refreshed every 72 h, with the same type and concentration of exosomes replenished in an equal volume at each medium change to maintain a stable exosome concentration in the culture system. In all experimental groups designated for miRNA regulation, we performed transfection with miRNA mimics or inhibitors in parallel with every medium change. The osteogenic induction medium was refreshed every 72 h.

Seven days following the initiation of osteogenic induction, we extracted the total RNA from the cells and analyzed it by quantitative real-time polymerase chain reaction (qRT-PCR). On this same day, we assayed ALP activity using an ALP detection kit (Biyuntian, Shanghai, China) strictly according to the manufacturer’s protocol; the absorbance of the reaction product in the cell culture supernatant was subsequently measured at a wavelength of 520 nm with a BioTek Epoch microplate reader (USA). Furthermore, ALP staining was conducted using a BCIP/NBT alkaline phosphatase chromogenic kit (Biyuntian, Shanghai, China) in accordance with the manufacturer’s protocol. Following 21 days of osteogenic induction, matrix mineralization capacity was assessed by means of Alizarin Red S (ARS, MW: 342.26 g/mol, ≥95.0% purity, Sigma-Aldrich, St. Louis, MO, USA) staining. Following fixation, cells were stained with 2% ARS solution. (pH 4.2; Sigma-Aldrich) at room temperature for 30 min, following the manufacturer’s protocol, followed by thorough washing with distilled water to eliminate non-specific staining signals. Micrographs of the mineralized nodules with positive staining were taken using an inverted light microscope (Olympus, Tokyo, Japan). For the quantitative assessment of mineralization, the stained nodules were incubated with 10% cetylpyridinium chloride (CPC, MW: 339.99 g/mol, ≥99.0% purity, Sigma-Aldrich, St. Louis, MO, USA) destaining solution (dissolved in 10 mM sodium phosphate buffer), and the absorbance of the resulting eluate was measured at 562 nm with a microplate reader.

### 2.9. Immunofluorescence Staining for Osteogenic Markers

To assess the stage-specific effects exerted by exosome treatment during osteogenic differentiation, key markers were analyzed at distinct time points corresponding to their peak expression during the differentiation process. The expression and subcellular localization of the early transcription factor RUNX2 were assessed at day 7 of osteogenic induction, which marks the commitment phase. Meanwhile, the production of the late-stage extracellular matrix protein Osteocalcin (OCN) was detected at day 14, during the matrix maturation and mineralization stage. For each target, immunofluorescence staining was performed as follows. We processed the cells in accordance with standard immunofluorescence protocols: first fixing them with 4% paraformaldehyde, then conducting cell permeabilization and non-specific binding blocking in sequence. Following the above procedures, overnight incubation of the cells was carried out at 4 °C in the presence of primary antibodies specific for RUNX2 (1:1000 dilution, GTX00792, GeneTex) or OCN (1:1000 dilution, DF12303, Affinity). After thorough rinsing to remove unbound antibodies, the corresponding Alexa Fluor-conjugated secondary antibodies (including Alexa Fluor 488 and 594) were supplemented, and the cells were incubated at room temperature for 1 h in the dark in accordance with the manufacturer’s protocol. 4′,6-diamidino-2-phenylindole (DAPI, MW: 350.25 g/mol, ≥98.0% purity, Sigma-Aldrich, St. Louis, MO, USA) was subsequently used for nuclear counterstaining, and fluorescent images were captured with confocal laser scanning microscopy (Olympus FV3000, Olympus Corporation, Tokyo, Japan). For fluorescence intensity quantification, at least three random fields of view per sample were analyzed using ImageJ software (v1.53, NIH); background fluorescence signals were deducted based on negative control groups where primary antibodies were omitted. All experimental procedures were carried out in triplicate.

### 2.10. qRT-PCR Analysis

We first isolated total cellular RNA using TRIzol reagent (Takara, Tokyo, Japan) by strictly following the manufacturer’s operating instructions. For the preparation of complementary DNA (cDNA) templates for subsequent assays, we reverse-transcribed the purified RNA into cDNA using the PrimeScript™ RT Master Mix kit (Takara, Kusatsu, Shiga, Japan), with all operational steps performed strictly in compliance with the official protocol provided by the manufacturer. For exosomal miRNA analysis, total miRNA was isolated using a Universal miRNA Extraction Kit (Tsinke Biotechnology Co., Ltd., Beijing, China), and the corresponding cDNA was then generated using the Goldenstar™ RT6 cDNA Synthesis Kit (Tsinke Biotechnology), both performed in accordance with the respective manufacturer’s guidelines. Quantitative real-time PCR (qRT-PCR) was conducted using an Applied Biosystems 7500 Real-Time PCR System (Thermo Fisher Scientific) with SYBR Green PCR Master Mix (Thermo Fisher Scientific, Waltham, MA, USA). The 20 μL reaction system was used for all assays, and the amplification program was set as follows: 95 °C pre-denaturation for 30 s; 40 cycles of 95 °C for 5 s and 60 °C for 34 s; followed by melt curve analysis: 95 °C for 15 s, 60 °C for 1 min, and gradual heating to 95 °C at 0.3 °C/s increments. We normalized the expression levels of mRNA and miRNA to the endogenous housekeeping genes glyceraldehyde-3-phosphate dehydrogenase (GAPDH) and U6 small nuclear RNA (U6), respectively. The relative expression of target genes was calculated via the 2^−ΔΔCt^ algorithm, with the sequences of all PCR primers provided in Supplementary Table S1.

### 2.11. Western Blot

We first extracted total cellular protein with RIPA lysis buffer (Zhonghuihecai, Beijing, China) with the addition of 1% (*v*/*v*) protease inhibitor cocktail. For each protein lysate, protein concentration was determined with a BCA protein assay kit, in strict accordance with the manufacturer’s instructions. Equal amounts of protein (20 μg per lane) were separated by SDS-PAGE with 4–20% gradient gels (EpiZyme Biotechnology, Shanghai, China) under constant voltage mode: 80 V for 30 min in the stacking gel, followed by 120 V for 60 min in the separating gel. Next, electrophoretic transfer was performed to move the separated proteins onto polyvinylidene difluoride (PVDF) membranes (0.22 μm pore size, Merck-Millipore, Darmstadt, Germany) under ice bath conditions with a constant current of 300 mA for 90 min. To block non-specific binding, PVDF membranes were incubated with 5% (*w*/*v*) non-fat milk (dissolved in TBST: 0.1% Tween-20 in Tris-buffered saline) at room temperature for 1 h. After the blocking step, the membranes were incubated with primary antibodies overnight at 4 °C that had been diluted in the above blocking solution. The primary antibodies applied in this experiment included: anti-GAPDH (1:5000, Cat. No. HRP-60004, Proteintech, Rosemont, IL, USA); anti-ALP (1:500, Cat. No. ab65834, Abcam, Cambridge, MA, USA); anti-RUNX2 (1:1000, Cat. No. GTX00792, GeneTex, Irvine, CA, USA); anti-COL1A1 (1:1000, Cat. No. 67288-1-Ig, Proteintech, Rosemont, IL, USA); anti-OCN (1:1000, Cat. No. DF12303, Affinity, Cincinnati, OH, USA); anti-TSG101 (1:1000, Cat. No. ab125011, Abcam, Cambridge, MA, USA); anti-CD63 (1:1000, Cat. No. 67605-1-Ig, Proteintech, Rosemont, IL, USA); anti-Flotillin-2 (1:1000, Cat. No. 66881-1-Ig, Proteintech, Rosemont, IL, USA); anti-Calnexin (1:1000, Cat. No. ab22595, Abcam, Cambridge, MA, USA); anti-Noggin (1:5000, Cat. No. 84283-5-RR, Proteintech, Rosemont, IL, USA) and beta-Actin (1:5000, Mouse T0022, Affinity, Cincinnati, OH, USA). The membranes were then washed thoroughly with TBST, followed by incubation with HRP-conjugated secondary antibodies (1:5000 dilution) at room temperature for 1 h. After an additional round of TBST washing, enhanced chemiluminescence (ECL) reagent was used to detect protein bands according to the manufacturer’s guidelines, and the resulting band images were acquired with a chemiluminescence imaging system (Bio-Rad, Hercules, CA, USA). We quantified the density of each individual protein band using ImageJ software (National Institutes of Health, Bethesda, MD, USA), and the expression levels of target proteins were normalized against GAPDH, which served as the internal reference protein in this assay.

### 2.12. ROS Detection and Oxidative Stress Assays

For the oxidative stress assay, the BMSCs in each aforementioned experimental group were treated with exosomes or PBS immediately after 6 Gy irradiation, followed by stationary incubation at 37 °C in a 5% CO_2_ atmosphere. Exosomes were added only once at the initial stage without additional replenishment during the 48 h culture period to avoid artificial interference with the dynamic detection of oxidative stress markers.

The ROS-scavenging capacity of different exosome preparations (BMSC-Fe_3_O_4_-Exos and BMSC-Fe_3_O_4_-SMF-Exos) and of miRNA mimics or inhibitors was assessed by measuring intracellular ROS levels and key oxidative stress markers. Intracellular ROS were detected using two distinct fluorescent probes: 2′,7′-dichlorodihydrofluorescein diacetate (DCFH-DA, MW: 487.29 g/mol, ≥98.0% purity, Abcam, Cambridge, UK) for total ROS and dihydroethidium (DHE, MW: 315.42 g/mol, ≥95.0% purity, Servicebio, Wuhan, China) specifically for superoxide anions (O_2_^−^). Total intracellular ROS levels were quantified using a DCFH-DA Cellular ROS Assay Kit (ab113851, Abcam, Cambridge, MA, USA) strictly in accordance with the manufacturer’s protocol. Similarly, intracellular superoxide anion levels were measured using a Dihydroethidium (DHE) Superoxide Anion Fluorescent Probe Kit (G1904-100T, Servicebio, Wuhan, Hubei, China) in full accordance with the manufacturer’s instructions. For both assays, cells were incubated with the respective probes at 37 °C for 30 min in the dark. After incubation, cells were processed for imaging: nuclei were counterstained with Hoechst 33342 (MW: 561.93 g/mol, ≥98.0% purity, Sigma-Aldrich, St. Louis, MO, USA), and fluorescence was observed using a confocal laser scanning microscope. For quantitative analysis, the fluorescence intensity from the captured confocal images was evaluated using ImageJ software (National Institutes of Health, Bethesda, MD, USA). The mean fluorescence intensity (MFI) from at least three random fields per sample was calculated and used to represent the relative intracellular ROS levels.

To explore the mechanism by which exosomes alleviate oxidative stress, antioxidant enzyme activity, superoxide dismutase (SOD) and the content of lipid peroxidation product malondialdehyde (MDA) were measured. SOD activity was measured using a commercial SOD assay kit (GM1133, Servicebio, Wuhan, Hubei, China), and MDA content was assessed using a Malondialdehyde (MDA) Detection Kit (G4300-48T, Servicebio**,** Wuhan, Hubei, China); both assays were performed strictly according to the corresponding manufacturers’ protocols.

### 2.13. Dual-Luciferase Reporter Assay

The direct targeting association between miR-429 and the Noggin (NOG) gene was further confirmed using a dual-luciferase reporter system. The wild-type (WT) sequence of the NOG 3′ untranslated region (3′-UTR), which contained the predicted miR-429 binding site, was inserted into the pmirGLO dual-luciferase reporter vector (Promega, Madison, WI, USA). A corresponding mutant (MUT) reporter vector, with site-directed mutations in the binding site, was generated as a control. HEK-293T cells were seeded in 24-well plates and co-transfected using an appropriate transfection reagent. Each well received either the WT or MUT reporter vector, together with either a miR-429 mimic or a negative control (NC) mimic. The pRL-TK vector (Promega), expressing Renilla luciferase, was included in all transfections for normalization. After 48 h, cells were harvested and lysed, and luciferase activities were detected. We sequentially measured the activities of Firefly and Renilla luciferase with the Dual-Luciferase Reporter Assay System (Promega, Madison, WI, USA) via a luminometer, in strict compliance with the manufacturer’s operating guidelines. The relative luciferase activity was calculated as the ratio of Firefly luciferase activity to Renilla luciferase activity.

### 2.14. Cell Transfection

To regulate the expression of miR-429 and its presumptive target gene Nog, BMSCs were transfected with Lipofectamine 3000 reagent (Invitrogen, Waltham, MA, USA) according to the manufacturer’s protocol.

For the purpose of gain- and loss-of-function studies, transfection of BMSCs was performed using miR-429 mimic (50 nM) or miR-429 inhibitor (100 nM), along with their respective negative controls (miR-NC mimic and inhibitor NC; Tsingke Biotechnology, Beijing, China).

To overexpress the Nog gene, BMSCs were transfected with a Nog-overexpression plasmid (Tsingke Biotechnology, China) using the same transfection reagent. An empty vector was transfected in parallel as the control.

Forty-eight hours post-transfection, transfection efficiency was confirmed by quantifying the intracellular levels of miR-429 (for mimic/inhibitor transfection) or Nog mRNA (for plasmid transfection) via qRT-PCR. Subsequently, the cells subjected to these transfections were used for downstream functional assays and molecular analyses as described in the respective experimental sections.

### 2.15. Statistical Analysis

Data were obtained from a minimum of three independent biological replicates and presented as the mean ± standard deviation (SD). Statistical differences between the two groups were analyzed using a two-tailed Student’s *t*-test. When comparing three or more groups, one-way analysis of variance (ANOVA) was applied, followed by Tukey’s post hoc test for pairwise comparisons. All statistical evaluations were conducted using GraphPad Prism 7.0 software (GraphPad Software, San Diego, CA, USA). A *p*-value < 0.05 was regarded as statistically significant, and the levels of significance were denoted as: * *p* < 0.05, ** *p* < 0.01, *** *p* < 0.001, **** *p* < 0.0001.

## 3. Results

### 3.1. Characterization of Fe_3_O_4_ MNPs

**Figure 1:**
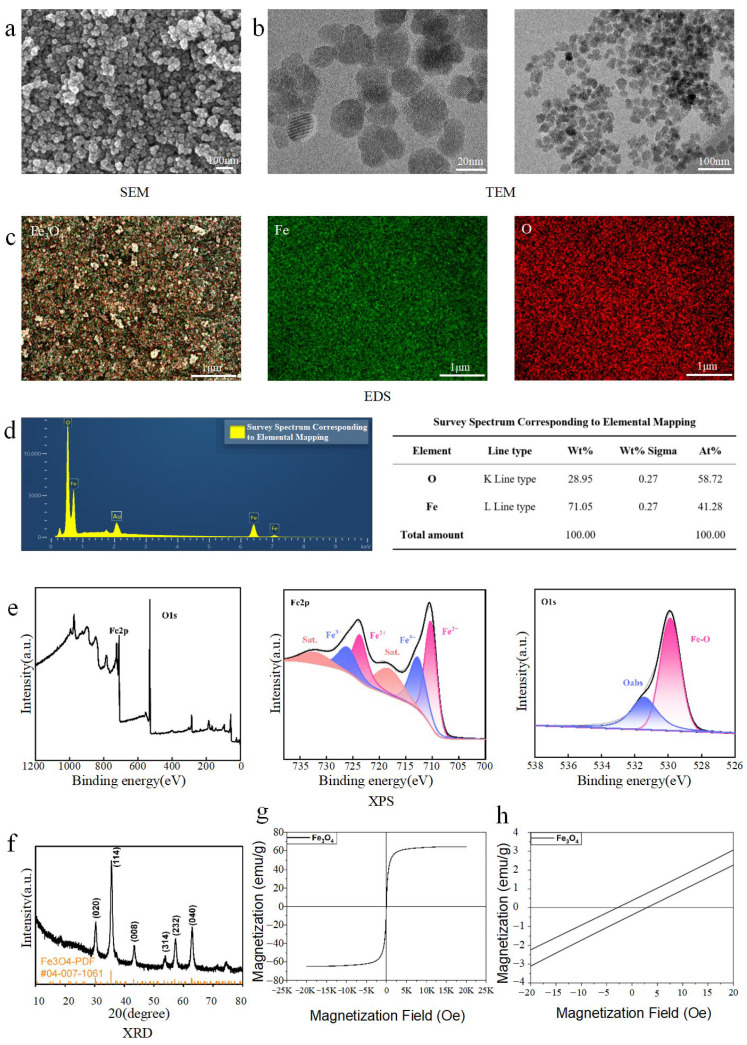
Characterization of Fe_3_O_4_ NPs. (**a**) SEM images of Fe_3_O_4_ NPs. (**b**) TEM images of Fe_3_O_4_ NPs. (**c**) EDS mapping images of Fe_3_O_4_ NPs, showing uniform distribution of Fe (red signal) and O (green signal) elements. (**d**) EDS full survey spectrum corresponding to the elemental mapping of Fe_3_O_4_ MNPs. Characteristic peaks of Fe (Lα ~0.7 keV, Kα ~6.4 keV, Kβ ~7.1 keV) and O (Kα ~0.52 keV) confirm the elemental composition of Fe_3_O_4_; the minor peak at ~2.12 keV corresponds to the Au Mα line, originating from the gold sputter coating for sample conductivity enhancement during SEM/EDS testing. (**e**) XPS spectra of Fe 2p and O 1s in Fe_3_O_4_ NPs. High-resolution Fe 2p spectrum shows distinct peaks for Fe^2+^ (710.2 eV, 723.8 eV) and Fe^3+^ (711.9 eV, 725.4 eV) (confirming Fe_3_O_4_ mixed valence state); O 1s spectrum is deconvoluted into lattice oxygen (O_2_^−^, 529.9 eV) and surface-adsorbed hydroxyl groups (OH, 531.5 eV). (**f**) XRD patterns of Fe_3_O_4_ NPs, matching the standard magnetite phase (JCPDS No. 19-0629) with no significant impurity peaks. (**g**,**h**) Magnetization curves and magnetization behavior at a magnetic field (−20–20 Oe) of Fe_3_O_4_ NPs, exhibiting typical superparamagnetic behavior with a saturation magnetization (Ms) of 64.55 emu/g, low coercivity (Hc), and minimal remanence (Mr).

The synthesized Fe_3_O_4_ MNPs were first characterized for their physicochemical properties. Scanning electron microscopy (SEM) micrographs revealed spherical particles assembled into dense aggregates (Figure 1a). Transmission electron microscopy (TEM) further showed that the individual MNPs were near-spherical or irregular in shape, with a diameter between 10 and 20 nm, clear boundaries, and observable crystallinity, though some agglomeration was present (Figure 1b). Statistical analysis of the particle size distribution from TEM images (*n* = 36) showed that the Fe_3_O_4_ MNPs had an average diameter of 21.54 ± 6.40 nm, with a narrow Gaussian distribution. This result was highly consistent with the hydrodynamic size measured by DLS (Z-average: 34.82 nm), considering the inherent difference between the dry-state physical diameter (TEM) and the hydrated hydrodynamic diameter (DLS), further confirming the excellent dispersity and uniform size of the synthesized MNPs. The corresponding particle size distribution histogram is provided in the Supplementary Materials as Figure S2. Energy dispersive X-ray spectroscopy (EDS) characterization verified the elemental constituents and purity of the synthesized Fe_3_O_4_ MNPs. The full survey spectrum (Figure 1d) showed dominant characteristic peaks of Fe and O, with the atomic percentages of iron and oxygen measured at 41.28% and 58.72%, respectively, which is highly consistent with the theoretical stoichiometric ratio of Fe_3_O_4_. The minor peak at ~2.12 keV was identified as the Au Mα line, which was introduced by the gold sputter coating during sample preparation to enhance electrical conductivity for SEM imaging, with no contribution to the elemental composition of the synthesized Fe_3_O_4_ MNPs. The corresponding elemental mapping images (Figure 1c) further confirmed the uniform distribution of Fe and O elements throughout the nanoparticles, with no heterogeneous impurity regions observed. X-ray photoelectron spectroscopy (XPS) was further employed to analyze the elemental composition and chemical states of the synthesized Fe_3_O_4_ MNPs. The survey scan spectrum revealed characteristic photoelectron peaks corresponding to Fe 2p, O 1s, and C 1s (adventitious carbon), with no detectable signals from other elements such as Si or Al, confirming the high chemical purity of the synthesized nanoparticles. High-resolution XPS spectra of Fe 2p were deconvoluted to reveal the mixed valence states characteristic of Fe_3_O_4_. In the Fe 2p spectrum, two distinct peaks at approximately 710.2 and 723.8 eV were assigned to Fe^2+^ 2p_3_/_2_ and Fe^2+^ 2p_1_/_2_, whereas those at 711.9 and 725.4 eV corresponded to Fe^3+^ 2p_3_/_2_ and Fe^3+^ 2p_1_/_2_, respectively. The absence of a distinct satellite peak at around 719 eV, which is typically associated with γ-Fe_2_O_3_, further supports the formation of the Fe_3_O_4_ phase. The O 1s spectrum was deconvoluted into two components: a main peak at 529.9 eV attributed to lattice oxygen (O^2−^) in Fe_3_O_4_, and a smaller peak at 531.5 eV assigned to surface-adsorbed hydroxyl groups (OH) or oxygen vacancies. Quantitative analysis based on peak areas yielded an Fe/O atomic ratio of approximately 0.71, which is close to the theoretical stoichiometric ratio of Fe_3_O_4_ (0.75), further confirming the successful synthesis of phase-pure Fe_3_O_4_ MNPs (Figure 1e) [37]. X-ray diffraction (XRD) patterns of the MNPs corresponded well with the standard magnetite phase [38], and no significant impurity peaks were detected, indicating high phase purity (Figure 1f). The XRD-derived crystallite size (18.4 nm) was slightly smaller than the TEM-measured particle size (21.54 ± 6.40 nm, n = 36), which is reasonable as XRD reflects crystalline domain size while TEM measures overall particle size (including surface coating/agglomeration). To further verify the colloidal stability of the synthesized Fe_3_O_4_ MNPs in aqueous solution, we performed dynamic light scattering (DLS) and zeta potential measurements. DLS results showed that the Z-average hydrodynamic diameter of Fe_3_O_4_ MNPs was 34.82 nm with a polydispersity index (PI) of 0.1266, and the median diameter (D_50_) was 37.92 nm, which was highly consistent with the particle size range observed by TEM, indicating no severe agglomeration of the nanoparticles in the aqueous phase and a narrow size distribution. Zeta potential analysis revealed that the Fe_3_O_4_ MNPs had an average zeta potential of −29.38 mV in ultrapure water. The absolute value of the zeta potential exceeded 25 mV, which meets the widely recognized standard for good colloidal stability of nanoparticle dispersions, confirming that the synthesized Fe_3_O_4_ MNPs can maintain a stable and uniform dispersion state in aqueous solution. This property effectively avoids particle agglomeration during co-culture with BMSCs, ensures the stability of the actual acting concentration of MNPs, and lays a reliable foundation for the repeatability and reliability of all subsequent cell experiments. The hydrodynamic size and zeta potential distribution diagrams are shown in Figure S1 (Supplementary Materials). The magnetization curve indicated that the synthesized Fe_3_O_4_ MNPs exhibited a high saturation magnetization (Ms) of 64.55 emu/g, along with typical superparamagnetic/soft magnetic behavior characterized by low coercivity (Hc) and minimal remanence (Mr). The high Ms value ensures strong magnetic responsiveness under an applied magnetic field, while the low Mr contributes to reduced magnetic aggregation in biological environments (Figure 1g,h) [39].

**Figure 2:**
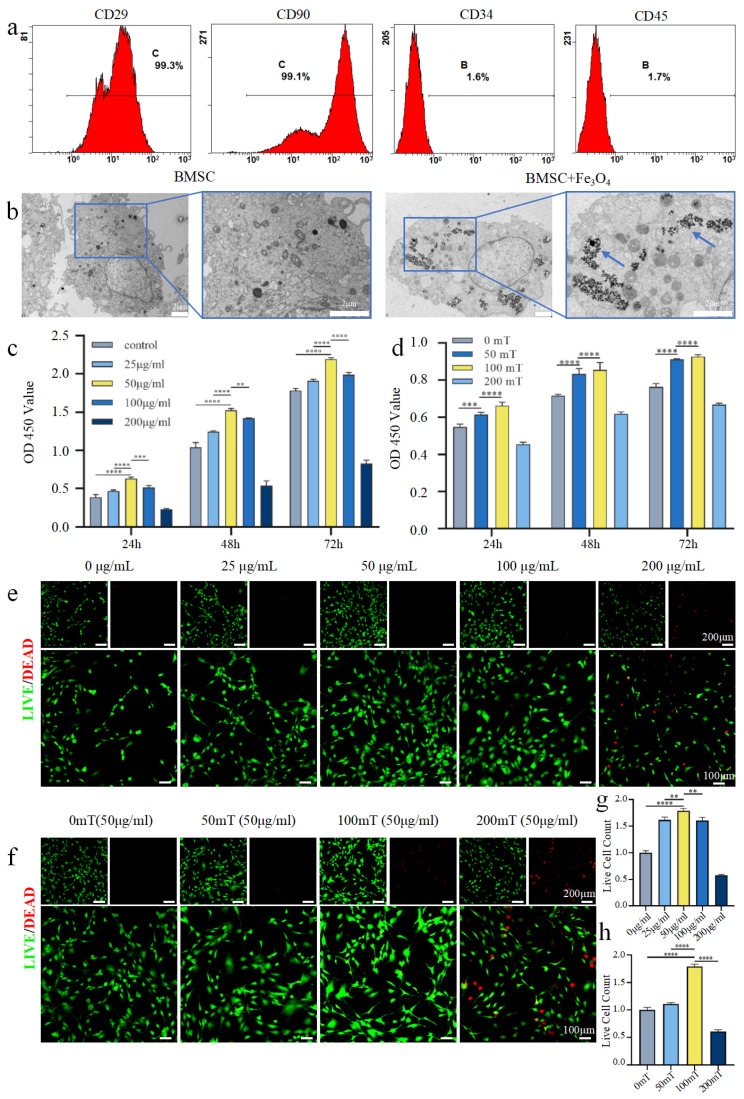
In Vitro Biocompatibility of Fe_3_O_4_ NPs. (**a**) Immunophenotypic characterization of BMSCs via flow cytometry, showing high positivity for mesenchymal markers CD29 and CD90 and low expression of hematopoietic markers CD34 and CD45. (**b**) TEM images showing the internalization of Fe_3_O_4_ NPs in BMSCs, with blue arrows indicating Fe_3_O_4_ NPs. (**c**) CCK-8 assay was performed to assess the proliferation of BMSCs treated with different concentrations of Fe_3_O_4_ NPs (0, 25, 50, 100, 200 μg/mL), with the ordinate representing OD_450_ value (cell proliferation indicator) and abscissa representing culture time (24 h, 48 h, 72 h). (**d**) Proliferation of BMSCs treated with the optimal concentration (50 μg/mL) of Fe_3_O_4_ NPs and exposed to SMF of different strengths (0, 50, 100, 200 mT), with the ordinate representing OD_450_ value and abscissa representing culture time (24 h, 48 h, 72 h). (**e**,**g**) Live/dead cell staining fluorescence images (green: live cells, red: dead cells; scale bar = 200 μm) and quantification of BMSCs co-cultured with Fe_3_O_4_ at various concentrations (0, 25, 50, 100, 200 μg/mL) for 72 h, with the ordinate of quantification graphs representing relative live cell count. (**f**,**h**) Live/dead cell staining fluorescence images (green: live cells, red: dead cells; scale bar = 200 μm) and quantification of BMSCs co-cultured with 50 μg/mL Fe_3_O_4_ NPs and different SMF intensities (0, 50, 100, 200 mT) for 72 h, with the ordinate of quantification graphs representing relative live cell count. n = 3 (**) *p* < 0.005. (***) *p <* 0.001. (****) *p* < 0.0001.

Surface marker expression of the cultured BMSCs was analyzed by flow cytometry. The results demonstrated high positivity for the characteristic mesenchymal markers CD29 (99.3%) and CD90 (99.1%), while the expression of the hematopoietic lineage markers CD34 and CD45 was negligible (1.6% and 1.7%, respectively). This immunophenotypic profile is consistent with the established criteria for defining MSCs (Figure 2a).

Transmission electron microscopy examination of BMSCs treated with 50 μg/mL iron tetroxide confirmed that the MNPs were successfully internalized by the cells. These MNPs appeared as electron-dense particles, distributed in the cytoplasmic compartments, and did not affect the normal cell morphology (Figure 2b).

To establish the optimal biocompatible conditions for subsequent exosome generation, we screened the effects of Fe_3_O_4_ nanoparticle concentration and SMF intensity on BMSCs viability. To assess the biocompatibility of the MNPs and determine their optimal working concentration, BMSCs were co-cultured with Fe_3_O_4_ NPs at concentrations of 0, 25, 50, 100, and 200 μg/mL. The CCK-8 assay results (Figure 2c) showed that the cell proliferation rate in the 50 μg/mL group was significantly higher than that in all other concentration groups throughout the culture period (n = 3 *p* < 0.005), while the 200 μg/mL group exhibited the lowest proliferative activity. These findings were further corroborated by live/dead cell staining (Figure 2e,g). After 72 h of culture, the 50 μg/mL group demonstrated the greatest density of viable cells and the fewest dead cells, indicating optimal cytocompatibility at this concentration. Consequently, 50 μg/mL was selected as the working concentration for the following experiments.

To explore the biological effects of static magnetic field (SMF) on BMSCs, cells pretreated with 50 μg/mL Fe_3_O_4_ NPs were exposed to SMF at intensities of 0, 50, 100, and 200 mT. The CCK-8 proliferation assay (Figure 2d) revealed that the cell proliferation rate in the 100 mT group was significantly higher than those in the other magnetic field intensity groups at all detected time points. (n = 3, *p* < 0.0001). Consistent with this, live/dead staining images (Figure 2f,h) displayed the greatest number of viable cells and the fewest dead cells in the 100 mT group.

In summary, the experimental data indicated that the combined treatment of 50 μg/mL NPs and a 100 mT SMF most effectively promoted the proliferation and survival of BMSCs without compromising cellular activity. Based on these results, this combination was established as the in vitro treatment condition for subsequent osteogenic differentiation induction of BMSCs.

### 3.2. Characterization and Internalization of Exosomes

**Figure 3:**
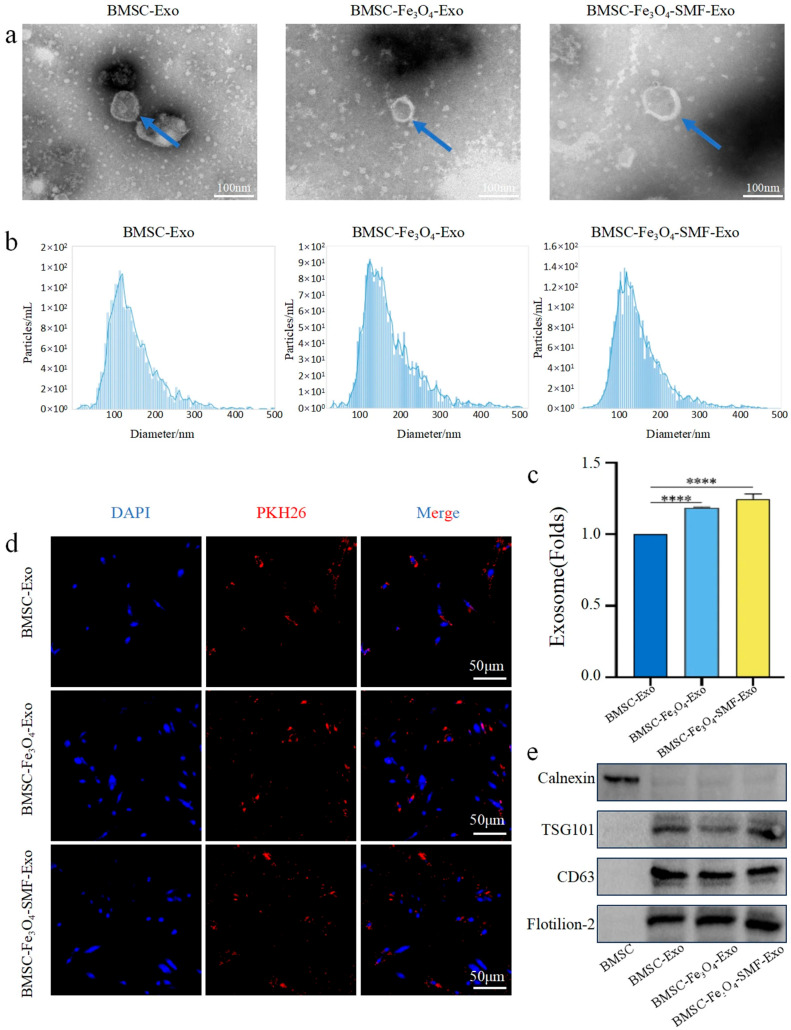
Characterization and internalization assessment of three distinct exosome subtypes. (**a**) TEM images showing the morphological features of BMSC-Exo, BMSC-Fe_3_O_4_-Exo, and BMSC-Fe_3_O_4_-SMF-Exo; blue arrows indicate exosomes with typical cup-shaped or spherical morphology (scale bar = 100 nm). (**b**) Nanoparticle tracking analysis (NTA) of the three exosome subtypes, revealing similar size distributions predominantly within the 50–150 nm exosomal range (abscissa: Diameter/nm; ordinate: Particles/mL). (**c**) Quantitative comparison of exosome production, showing that Fe_3_O_4_ NPs combined with SMF significantly increase exosome yield in BMSCs; the ordinate represents exosome relative fold change compared to BMSC-Exo (*p* < 0.0001). (**d**) Uptake of three exosome subtypes labeled with red fluorescent dye PKH26 by BMSCs; confocal images show DAPI-stained nuclei (blue), PKH26-labeled exosomes (red), and merged images (scale bar = 50 μm), demonstrating effective internalization with punctate cytoplasmic distribution. (**e**) Western blotting analysis of exosomal marker proteins CD63, TSG101, and Flotillin-2 (positive expression confirms exosomal identity) and the endoplasmic reticulum contaminant protein Calnexin (negative expression indicates high exosome purity) in BMSCs and the three exosome subtypes. n = 3 (****) *p* < 0.0001.

Next, we characterized the three isolated exosome subpopulations—BMSC-Exos, BMSC-Fe_3_O_4_-Exos, and BMSC-Fe_3_O_4_-SMF-Exos—via transmission electron microscopy (TEM), nanoparticle tracking analysis (NTA), and Western blotting. TEM images confirmed that all three subtypes exhibited the typical cup-shaped or spherical morphology of exosomes, with no apparent differences in their ultrastructural appearance (Figure 3a). NTA revealed that the particle size distributions of all groups were predominantly within the characteristic exosomal range of 50–150 nm (Figure 3b). Quantification of particle concentration indicated that the yields of both BMSC-Fe_3_O_4_-Exos and BMSC-Fe_3_O_4_-SMF-Exos were markedly increased compared with those of conventional BMSC-Exos. Furthermore, the BMSC-Fe_3_O_4_-SMF-Exos group yielded a greater number of particles compared to the BMSC-Fe_3_O_4_-Exos group (n = 3 *p* < 0.0001) (Figure 3c). Western blot analysis further validated the exosomal identity, as all three preparations positively expressed the specific markers Flotillin-2, CD63, and TSG101, while testing negative for the endoplasmic reticulum contaminant protein Calnexin (Figure 3e).

To determine the endocytic internalization of exosomes by BMSCs, exosomes derived from each group were initially labeled with the red fluorescent probe PKH26 (Sigma-Aldrich, Darmstadt, Germany) and then co-incubated with BMSCs for 24 h. Fluorescence microscopy demonstrated that all three exosome subtypes were effectively internalized by recipient BMSCs, displaying a punctate cytoplasmic distribution (Figure 3d).

### 3.3. BMSC-Fe_3_O_4_-SMF-Exos Promote Osteogenic Differentiation of Irradiated BMSCs in Vitro

**Figure 4:**
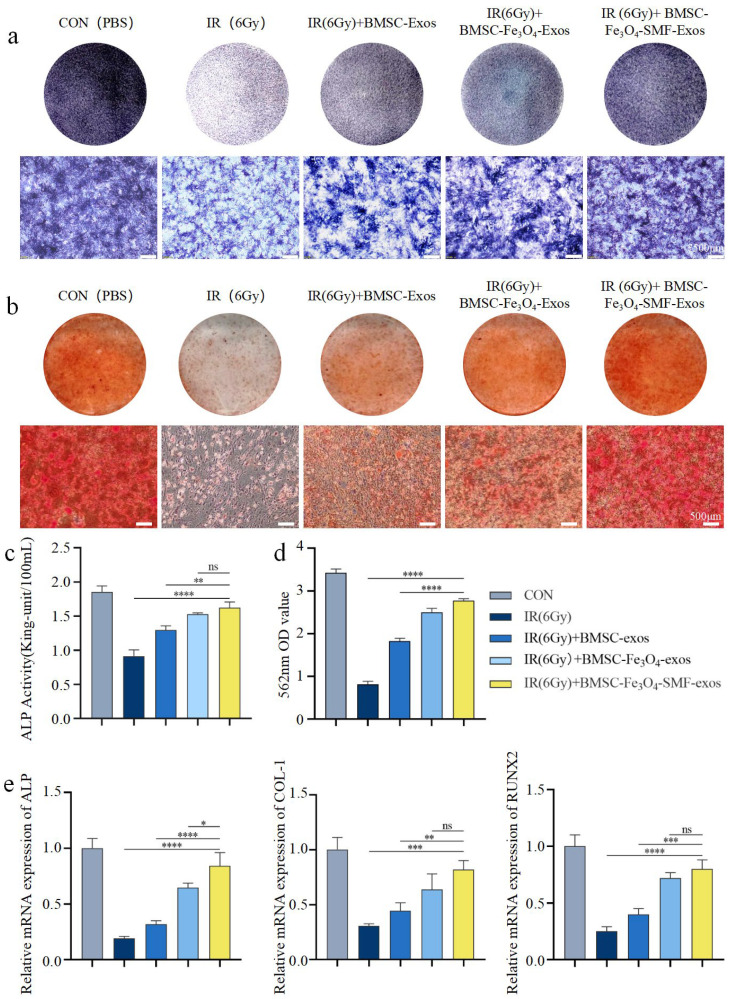
BMSC-Fe_3_O_4_-SMF-Exos Facilitate Osteogenic Differentiation of Irradiated BMSCs In Vitro. (**a**,**c**) Following 7 days of co-culture with exosomes, ALP staining and the associated quantitative analysis were conducted in irradiated BMSCs. (**b**,**d**) Following 21 days of exosome co-culture, ARS staining was performed to assess the osteogenic differentiation potential of irradiated BMSCs, with quantitative analysis performed simultaneously to determine the staining intensity. (**e**) Quantitative real-time PCR (qRT-PCR) analysis of osteogenic marker gene expression, showing relative mRNA levels of RUNX2 (osteogenic transcription factor), COL1A1 (collagen type I alpha 1 chain), and ALP (alkaline phosphatase); all exosome groups upregulate gene expression compared to IR group, with BMSC-Fe_3_O_4_-SMF-Exo group showing the most significant upregulation. n = 3 (*) *p* < 0.05; (**) *p* < 0.01; (***) *p* < 0.001; and (****) *p* < 0.0001; ns: not significant.

BMSCs were irradiated with 6 Gy and subsequently underwent osteogenic induction. On day 7 post-irradiation, ALP staining and activity quantification were performed. The IR group showed markedly suppressed ALP activity. All three types of exosomes restored the alkaline phosphatase (ALP) activity in the irradiated BMSCs. Notably, the BMSC-Fe_3_O_4_-SMF-exosome group exhibited the strongest staining intensity and the highest quantitative value, indicating the most potent pro-osteogenic effect (n = 3 *p* < 0.005) (Figure 4a,c).

To assess late-stage osteogenic maturation, ARS staining for mineralized nodule formation was conducted on day 21. Consistent with the ALP findings, irradiation severely impaired matrix mineralization, as evidenced by significantly reduced ARS staining in the IR group (n = 3 *p* < 0.0001). Exosome treatments mitigated this impairment, with the BMSC-Fe_3_O_4_-SMF-Exos group exhibiting the most extensive and intense red staining, indicating the highest degree of calcium deposition among all treatment groups (Figure 4b). Quantitative analysis of eluted ARS further confirmed that, compared with the irradiation-only group, BMSC-Fe_3_O_4_-SMF-Exos significantly rescued irradiation-induced mineralization deficits (n = 3 *p* < 0.0001) (Figure 4d).

Consistent with this, qRT-PCR analysis demonstrated that irradiation significantly downregulated the expression of key osteogenic genes (COL1A1, RUNX2, ALP). All exosome treatments upregulated their expression compared to the IR group. Notably, BMSC-Fe_3_O_4_-SMF-Exos induced the most pronounced upregulation of these transcripts, significantly outperforming both conventional BMSC-Exos and BMSC-Fe_3_O_4_-Exos (n = 3 *p* < 0.005) (Figure 4e). Immunofluorescence staining and quantitative analysis for key markers were performed at early (day 7) and late (day 14) stages post-induction.

**Figure 5:**
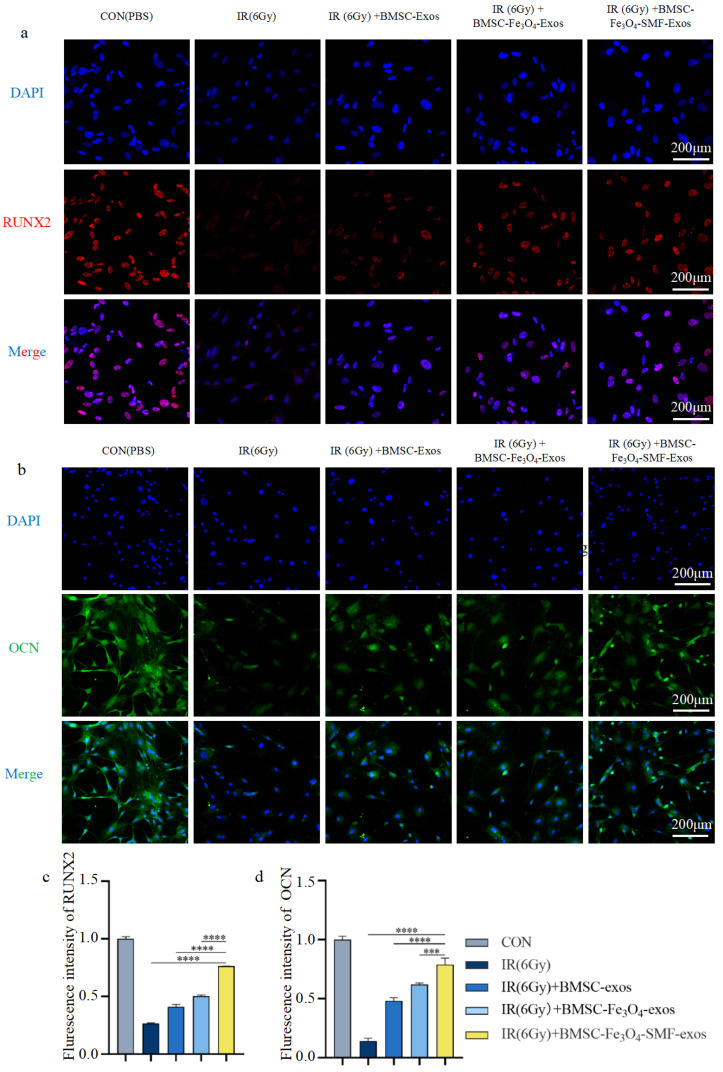
BMSC-Fe_3_O_4_-SMF-Exos Promote Osteogenic Differentiation of Irradiated BMSCs In Vitro (**a**) Representative immunofluorescence images showing the expression of the early osteogenic transcription factor RUNX2 (red) in BMSCs on day 7 post-irradiation. (**b**) Quantitative analysis of the mean fluorescence intensity (MFI) for RUNX2 on day 7. (**c**) Representative immunofluorescence images showing the expression of the late osteogenic marker Osteocalcin (OCN, green) in BMSCs on day 14 post-irradiation. Nuclei were counterstained with DAPI (blue). Scale bar: 200 μm. (**d**) Quantitative analysis of the mean fluorescence intensity (MFI) of OCN on day 14. n = 3; (***) *p* < 0.001, and (****) *p* < 0.0001.

Detection of the early core transcription factor RUNX2 on day 7 revealed that irradiation significantly suppressed its expression (n = 3 *p* < 0.0001). All exosome treatment groups promoted the recovery of RUNX2 expression. Among them, the BMSC-Exos group showed partial restoration, the BMSC-Fe_3_O_4_-Exos group exhibited more substantial recovery, while the BMSC-Fe_3_O_4_-SMF-Exos group demonstrated the strongest restorative effect (Figure 5a). Further quantitative analysis of fluorescence intensity confirmed that the upregulation of RUNX2 by BMSC-Fe_3_O_4_-SMF-Exos was significantly superior to that of the other exosome treatment groups (n = 3 *p* < 0.0001) (Figure 5c).

Detection of the late functional protein OCN on day 14 further elucidated the maturation stage of differentiation. Irradiation similarly severely inhibited OCN expression (n = 3 *p* < 0.0001). All exosome treatments upregulated OCN to varying degrees, with the BMSC-Fe_3_O_4_-SMF-Exos group showing the most pronounced promotive effect (Figure 5b). Quantitative results verified that BMSC-Fe_3_O_4_-SMF-Exos was significantly more effective than the other control groups in enhancing OCN expression levels (n = 3 *p* < 0.001) (Figure 5d).

Taken together, these results demonstrate a clear efficacy hierarchy: BMSC-Fe_3_O_4_-SMF-Exos manifested the most remarkable activity in rescuing the irradiation-impaired osteogenic differentiation of BMSCs, which was significantly higher than that in the other two groups.

### 3.4. BMSC-Fe_3_O_4_-SMF-Exos Attenuate Intracellular ROS in Irradiated BMSCs

To investigate the regulatory effects of different exosomes on intracellular ROS levels following radiation, BMSCs were treated immediately after exposure to 6 Gy of irradiation. Their antioxidant effects were systematically evaluated using fluorescent probes and biochemical assays.

First, intracellular ROS levels were assessed using the DCFH-DA probe, which emits green fluorescence upon oxidation by ROS, with fluorescence intensity proportional to ROS concentration.

**Figure 6:**
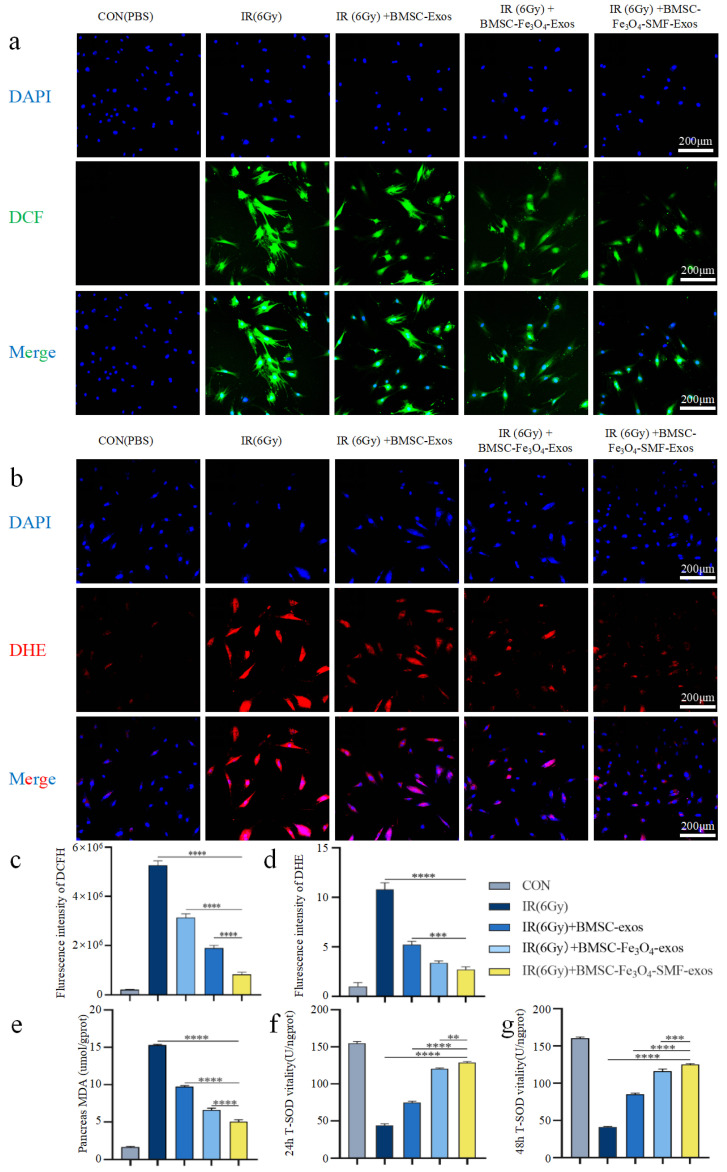
BMSC-Fe_3_O_4_-SMF-Exos Mitigate Intracellular ROS in Irradiated BMSCs. (**a**,**c**) Intracellular total ROS levels in BMSCs were evaluated utilizing the 2′,7′-dichlorodihydrofluorescein diacetate (DCF-DA) fluorescent probe, accompanied by the relevant quantitative determination. (**b**,**d**) Superoxide anion (O_2_^−^) production in irradiated BMSCs was monitored with the DHE fluorescent probe, and concurrent quantitative analysis was implemented. (**e**) The concentration of MDA in irradiated BMSCs was quantified. (**f**,**g**) The levels of SOD in irradiated BMSCs were measured at the 24 h and 48 h time points, separately. n = 3; (**) *p* < 0.01; (***) *p* < 0.001, and (****) *p* < 0.0001.

As illustrated in Figure 6a, relative to the non-irradiated control group, the IR group displayed a pronounced elevation in green fluorescence, indicating a surge in ROS. Administration of BMSC-Fe_3_O_4_-Exos, and more profoundly with BMSC-Fe_3_O_4_-SMF-Exos, significantly suppressed this fluorescence, demonstrating superior ROS scavenging capacity over conventional BMSC-Exos. Quantitative analysis (Figure 6c) revealed that all three exosome treatment groups reduced the green fluorescence intensity compared to the IR group, with the effect being the most pronounced in the BMSC-Fe_3_O_4_-SMF-exosome group (n = 3 *p* < 0.0001).

Subsequently, the superoxide-sensitive probe DHE was used for validation, which yields red fluorescence upon oxidation. Confocal imaging (Figure 6b) illustrated the peri-nuclear distribution of ROS (red) relative to nuclei (blue). The results demonstrated that the three kinds of exosomes all obviously diminished the red fluorescence signal, with BMSC-Fe_3_O_4_-SMF-Exos being the most effective (Figure 6c). Quantitative DHE data (Figure 6d) corroborated these findings, showing the greatest reduction in the fluorescent signal intensity in the BMSC-Fe_3_O_4_-SMF-Exos group, which was markedly superior to other exosome treatments (n = 3 *p* < 0.001).

To uncover the potential mechanism through which the aforementioned exosomes alleviate oxidative stress, we further examined the dynamic changes in the activity of superoxide dismutase (SOD), a major antioxidant enzyme, and the accumulation level of the end-product of lipid peroxidation, malondialdehyde (MDA).

The results showed that irradiation significantly disrupted the cellular redox homeostasis. In the IR group, the level of MDA, a hallmark of oxidative damage, was markedly upregulated (n = 3 *p* < 0.0001) (Figure 6e). Concurrently, the activity of the crucial antioxidant defense enzyme SOD was markedly suppressed at 24 h post-irradiation (n = 3 *p* < 0.0001) (Figure 6f). However, all exosome treatments effectively reversed these abnormalities. Notably, BMSC-Fe_3_O_4_-SMF-Exos showed the strongest and most favorable regulatory activity: it not only restored SOD activity most profoundly and at the earliest time point (24 h), but also sustained this enhanced effect at 48 h (n = 3 *p* < 0.001) (Figure 6g). Furthermore, this treatment exhibited the most potent inhibitory effect on MDA accumulation, significantly outperforming the other exosome treatment groups.

### 3.5. miR-429 Derived from BMSC-Fe_3_O_4_-SMF-Exos Inhibits NOG by Targeting Its 3′-UTR

**Figure 7:**
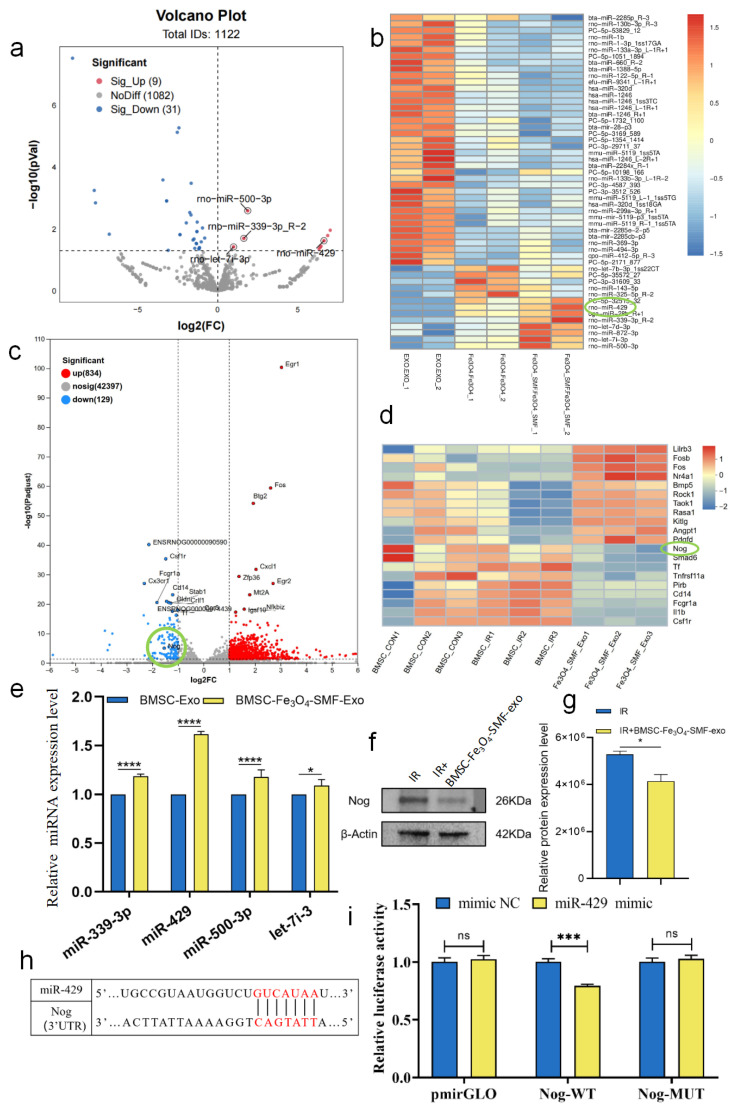
miR-429 derived from BMSC-Fe_3_O_4_-SMF-Exos inhibits NOG by targeting its 3′-UTR (wt stands for wild-type; mut refers to mutant; and NC represents negative control). (**a**,**b**) Heatmaps and volcano plots generated from miRNA sequencing analysis, where blue indicates downregulated miRNAs, red signifies upregulated counterparts, and the target miRNA is marked with a green circle; (**c**,**d**) Volcano plots and heatmaps were generated from transcriptome sequencing data, where blue represents downregulated mRNAs, red denotes upregulated mRNAs, and the target gene is marked with a green circle. (**e**) qRT-PCR analysis of four miRNAs in two exosomal populations. (**f**,**g**) Protein expression and quantitative analysis of NOG in irradiated BMSCs treated with two distinct exosomal populations. (**h**) Putative binding site of miR-429 situated in the 3′-UTR region of NOG; (**i**) Luciferase reporter activity in BMSCs co-transfected with either wild-type or mutant NOG 3′-UTR, along with control mimics or miR-429 mimics; n = 3 (*) *p* < 0.05; (***) *p* < 0.001, and (****) *p* < 0.0001; ns: not significant.

To explore the effects of various pretreatment strategies on the microRNA (miRNA) profile of exosomes derived from BMSCs, we performed small RNA sequencing on BMSC-Exos, BMSC-Fe_3_O_4_-Exos, and BMSC-Fe_3_O_4_-SMF-Exos. Analysis revealed an altered miRNA expression profile in BMSC-Fe_3_O_4_-SMF-Exos in comparison with BMSC-Exos. From this, we identified four miRNAs exhibiting the most significant upregulation: rno-miR-339-3p, rno-miR-429, rno-miR-500-3p, and rno-let-7i-3p (Figure 7a,b). Subsequent molecular validation by qPCR demonstrated that, among the four candidates, miR-429 was identified as the most markedly elevated miRNA in BMSC-Fe_3_O_4_-SMF-Exos relative to BMSC-Exos (n = 3 *p* < 0.001) (Figure 7e). A review of existing literature revealed that miR-429 promotes osteogenesis by protecting osteoblasts, primarily through alleviating oxidative stress and reducing ROS production [31]. Therefore, based on the aforementioned miRNA sequencing results and supporting literature, miR-429 was chosen as the primary candidate for subsequent research into the mechanisms by which BMSC-Fe_3_O_4_-SMF-Exos promote osteogenesis and mitigate oxidative stress.

Simultaneously, we performed transcriptomic sequencing on irradiated BMSCs treated with either PBS or BMSC-Fe_3_O_4_-SMF-Exos, yielding a set of differentially expressed genes. To identify potential downstream targets of miR-429, we screened for its candidate target genes by employing two specialized miRNA target prediction bioinformatics algorithms, TargetScan (version 8.0) and miRDB (version 6.0). These predicted targets were then intersected with the differentially expressed gene set obtained from the transcriptomic sequencing analysis. Analysis revealed that the NOG gene, which encodes Noggin—a key inhibitor of the bone morphogenetic protein (BMP) signaling pathway—was present within this intersection. (Figure 7c,d) Noggin is known to suppress osteogenic differentiation by antagonizing BMP signaling [40,41,42,43]. Correspondingly, WB analysis of the irradiated BMSCs revealed that treatment with BMSC-Fe_3_O_4_-SMF-exosomes resulted in decreased NOG protein levels compared to the IR group. (n = 3 *p* < 0.05) (Figure 7f,g).

To verify the direct interaction between miR-429 and NOG, a dual-luciferase reporter assay was performed. Introduction of the miR-429 mimic into BMSCs decreased luciferase activity as compared with the control mimic group. This directly demonstrated that miR-429 specifically binds to the 3′UTR of NOG to suppress its expression. (n = 3 *p* < 0.001) (Figure 7h,i).

### 3.6. miR-429 Knockdown Abrogates the Therapeutic Effects of BMSC-Fe_3_O_4_-SMF-Exos on Irradiated BMSCs

**Figure 8:**
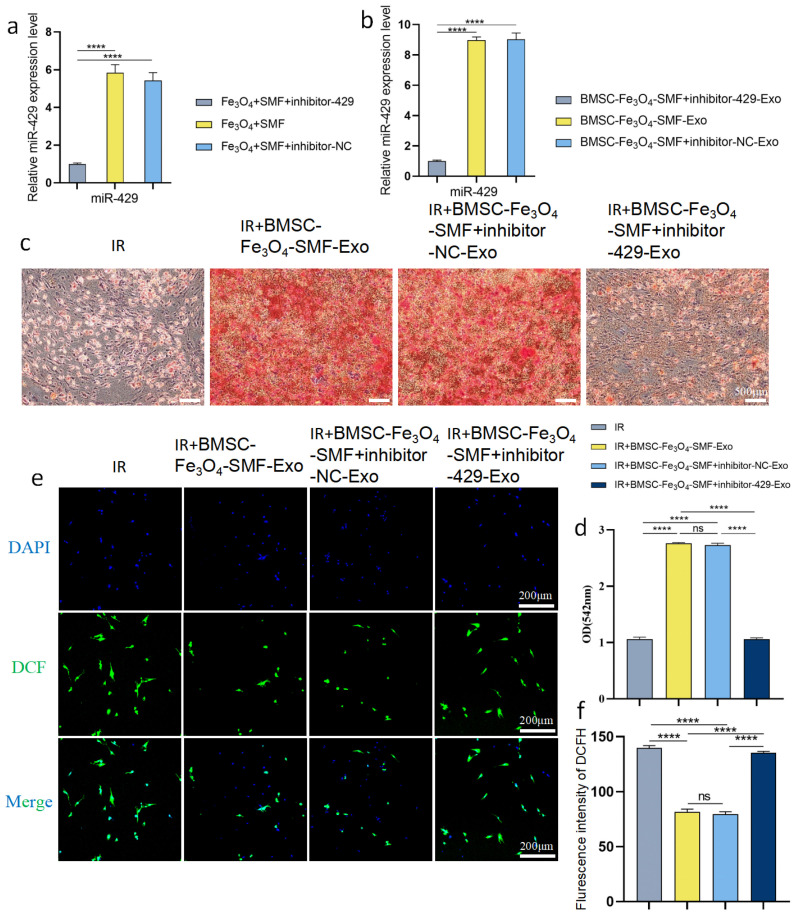
Loss-of-function experiments confirm that miR-429 mediates the therapeutic effects of BMSC-Fe_3_O_4_-SMF-Exos. (**a**) qRT-PCR analysis of miR-429 expression in preconditioned BMSCs transfected with miR-429 inhibitor, inhibitor-NC, or without transfection. (**b**) qRT-PCR analysis of miR-429 expression in exosomes derived from the above preconditioned BMSCs. (**c**) Representative Alizarin Red S (ARS) staining images of mineralized nodules in irradiated BMSCs after different treatments (scale bar = 500 μm). (**d**) Quantitative analysis of ARS staining absorbance at 562 nm to evaluate the level of extracellular matrix mineralization. (**e**) Representative fluorescence images of intracellular ROS detected by DCFH-DA staining (green: DCF, ROS indicator; blue: DAPI, nuclear staining; scale bar = 200 μm). (**f**) Quantitative analysis of DCF fluorescence intensity to assess intracellular ROS level. Statistical significance: (****) *p* < 0.0001; ns = not significant. n = 3.

To definitively confirm that the therapeutic effects of BMSC-Fe_3_O_4_-SMF-Exos are primarily mediated by miR-429, we constructed exosomes with miR-429 knockdown via transfecting miR-429 inhibitor into preconditioned BMSCs.qRT-PCR results showed that transfection of miR-429 inhibitor significantly reduced miR-429 expression in both preconditioned BMSCs (Figure 8a) and the derived exosomes (Figure 8b), while inhibitor-NC transfection had no significant effect on miR-429 expression, indicating the successful construction of miR-429-knockdown exosomes. We then evaluated the effect of miR-429-knockdown exosomes on the osteogenic differentiation of irradiated BMSCs. ARS staining showed that BMSC-Fe_3_O_4_-SMF-Exos and inhibitor-NC-Exos significantly promoted mineralized nodule formation in irradiated BMSCs, whereas this pro-osteogenic effect was completely abolished in the inhibitor-429-Exos group (Figure 8c). Quantitative analysis of ARS elution further confirmed this trend (Figure 8d). Meanwhile, we detected the effect of miR-429 knockdown on the antioxidant capacity of exosomes. DCFH-DA staining and quantitative analysis showed that BMSC-Fe_3_O_4_-SMF-Exos and inhibitor-NC-Exos significantly reduced intracellular ROS accumulation in irradiated BMSCs, while this antioxidant effect was completely reversed after miR-429 knockdown in exosomes (Figure 8e,f). Collectively, these loss-of-function results clearly demonstrated that miR-429 is the key functional mediator of BMSC-Fe_3_O_4_-SMF-Exos, and the therapeutic effects of the preconditioned exosomes on irradiated BMSCs are specifically attributable to miR-429, rather than other exosomal cargos.

### 3.7. miR-429 Promotes Osteogenic Differentiation and Reduces ROS Levels by Targeting and Inhibiting NOG

In the final step, in vitro rescue experiments were established to validate the regulatory role of the miR-429/NOG axis in osteogenesis and ROS clearance. Irradiated BMSCs were transfected with the following: mir-NC + pcDNA-NC, mir-429 mimics + pcDNA-NC, mir-NC + pcDNA-NOG, and mir-429 mimics + pcDNA-NOG.

**Figure 9:**
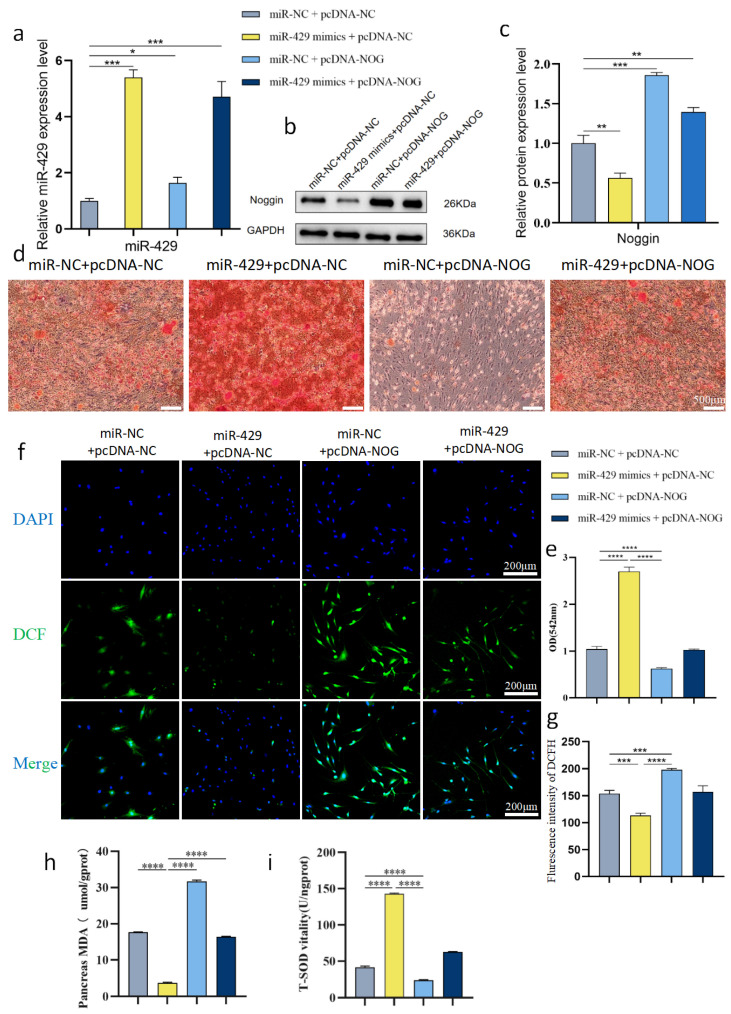
miR-429 promotes osteogenic differentiation and reduces ROS levels by targeting and inhibiting NOG to modulate the BMP/Smad pathway. (**a**) miRNA expression levels of miR-429 transfected with mir-NC + pcDNA-NC, mir-429 mimics + pcDNA-NC, mir-NC + pcDNA-NOG and mir-429 mimics + pcDNA-NOG in irradiated BMSCs; (**b**,**c**) Protein expression of NOG in irradiated BMSCs transfected with mir-NC + pcDNA-NC, mir-429 mimics + pcDNA-NC, mir-NC + pcDNA-NOG and mir-429 mimics + pcDNA-NOG; (**d**,**e**) Assessment of osteogenic differentiation via ARS staining and quantitative analysis in irradiated BMSCs following transfection and 21-day induction; (**f**,**g**) Measurement and quantitative analysis of total ROS levels in irradiated BMSCs from various transfection groups using the DCFH-DA fluorescent probe; (**h**) Measurement and quantitative analysis of total reactive oxygen species (ROS) levels in irradiated bone marrow-derived mesenchymal stem cells (BMSCs) from various transfection groups using the DCFH-DA fluorescent probe. (**i**) Measurement of SOD levels in irradiated BMSCs. n = 3 (*) *p* <0.05; (**) *p* < 0.01; (***) *p* < 0.001, and (****) *p* < 0.0001.

Following transfection, the relative expression level of intracellular miR-429 was determined by qPCR. The data revealed that, relative to the control group, miR-429 expression was markedly elevated in the miR-429 mimics + pcDNA-NC group, confirming the successful transfection efficiency of the mimics (n = 3 *p* < 0.001) (Figure 9a).

Western blot analysis revealed that the miR-429 overexpression group significantly reduced the protein expression level of NOG, whereas the miR-NC + pcDNA-NOG group exhibited a marked increase in NOG protein expression. Moreover, the co-transfection group (miR-429 + pcDNA-NOG) demonstrated that miR-429 could counteract the up-regulation of NOG, confirming an antagonistic interaction between groups (n = 3 *p* < 0.001). (Figure 9b,c).

To further validate this regulatory axis at the functional level, irradiated BMSCs were subjected to osteogenic induction for 21 days, followed by ARS staining. The staining results (Figure 9d) demonstrated that, compared with the control group (mir-NC + pcDNA-NC), the mir-429 mimics + pcDNA-NC group formed the highest number of mineralized nodules with the most intense staining, indicating the strongest mineralization capacity. Conversely, the mir-NC + pcDNA-NOG group showed barely detectable mineralized nodules, indicating a severe impairment in osteogenic differentiation. Notably, the osteogenic inhibition phenotype caused by NOG overexpression was significantly reversed in the mir-429 mimics + pcDNA-NOG group, with a partial restoration of mineralization capacity. Quantitative analysis of the ARS staining yielded results consistent with the morphological observations (n = 3, *p* < 0.0001) (Figure 9e).

Subsequently, oxidative stress levels in each group of cells were assessed. Using the DCFH-DA fluorescent probe to detect total intracellular ROS, fluorescence microscopy imaging (Figure 9f) revealed that the mir-429 mimics + pcDNA-NC group exhibited the weakest green fluorescence intensity, indicating the least intracellular ROS accumulation. In contrast, the mir-NC + pcDNA-NOG group displayed the strongest green fluorescence, suggesting significantly elevated ROS levels. The fluorescence intensity in the co-transfection group was intermediate, demonstrating that the introduction of miR-429 effectively counteracted the excessive ROS accumulation induced by NOG overexpression. Quantitative analysis of the fluorescence intensity corroborated these findings (n = 3 *p* < 0.001) (Figure 9g). Furthermore, biochemical markers associated with oxidative stress were measured. The results showed (n = 3, *p* < 0.0001) (Figure 9h,i) that the content of malondialdehyde (MDA), a terminal product of lipid peroxidation, was significantly increased in the mir-NC + pcDNA-NOG group, but markedly decreased in the mir-429 mimics + pcDNA-NC group. Conversely, the activity of the key antioxidant enzyme superoxide dismutase (SOD) exhibited an opposite trend: it was highest in the mir-429 mimics + pcDNA-NC group, yet significantly suppressed in the mir-NC + pcDNA-NOG group. In the co-transfection group, both the MDA content and SOD activity were restored to levels comparable to those of the control group. These consistent results indicate that miR-429 effectively alleviates oxidative stress in irradiated BMSCs, a protective effect that can be attenuated by NOG overexpression, thereby further confirming the molecular mechanism by which miR-429 exerts its antioxidant effect through targeted inhibition of NOG.

In summary, our data delineated a possible mechanistic pathway, in which BMSC-Fe_3_O_4_-SMF-Exos were enriched with miR-429 and delivered it into irradiated BMSCs. Within recipient cells, miR-429 directly bound to and suppressed NOG expression, thereby alleviating oxidative stress and restoring the radiation-impaired osteogenic differentiation process.

## 4. Discussion

Radiation-induced bone damage constitutes a severe and intractable complication associated with tumor radiotherapy [44], characterized by a complex pathological process characterized by sustained oxidative stress and impaired osteogenic differentiation of BMSCs. An optimal therapeutic strategy must concurrently alleviate oxidative damage and promote bone regeneration. Exosomes derived from stem cells have emerged as promising cell-free therapeutic agents due to their inherent regenerative and immunomodulatory properties [45,46]. However, their intrinsic biological efficacy is often insufficient to address multifactorial pathological conditions such as radiation-induced damage. Consequently, various engineering strategies—including hypoxic preconditioning, cytokine stimulation, and biomaterial-based priming—have been developed to enhance exosomal function [47,48,49]. Nevertheless, the therapeutic efficacy and mechanistic actions of such biomaterial-enhanced exosomes in radiation-induced bone injury remain poorly understood. To address this gap, this study innovatively employed a combined physical-material preconditioning approach, utilizing Fe_3_O_4_ MNPs in conjunction with an SMF to generate functionally enhanced exosomes (designated as BMSC-Fe_3_O_4_-SMF-Exos). For the first time, we systematically evaluated their therapeutic potential for treating radiation-induced bone injury. In this study, a 6 Gy irradiation dose was used to establish a radiation-induced bone injury cell model, a dose level widely recognized in previous studies for effectively inducing typical radiation damage in BMSCs without causing immediate widespread cell death. Importantly, 6 Gy mimics the cumulative effects of fractionated radiotherapy received by oral and maxillofacial bone tissue in clinical settings—consistent with the clinical context of radiation-associated dental and maxillofacial tissue injury emphasized by Brochado Martins et al. [50]—thereby replicating the core pathological features underlying radiation-induced bone injury, including suppressed osteogenesis and persistent oxidative stress [35,51]. Brochado Martins et al. further highlighted that radiation-induced damage to oral and maxillofacial tissues involves complex prognostic factors, and our 6 Gy in vitro model recapitulates the key cellular dysfunction (oxidative stress and impaired osteogenesis) observed in clinical radiation-induced bone injury, ensuring the clinical relevance of our experimental design.

Current strategies to enhance exosome efficacy are increasingly diverse. For instance, hypoxic preconditioning of parent cells could augment the pro-angiogenic and osteogenic capabilities of exosomes [52]. Electrical stimulation preconditioning has also been demonstrated to accelerate exosome-mediated bone regeneration. Among various nanomaterials, Fe_3_O_4_ MNPs have garnered significant attention due to their favorable biocompatibility, low systemic toxicity, and inherent osteoinductive properties. Studies indicated that Fe_3_O_4_ MNPs alone could promote the osteogenic differentiation of BMSCs; when combined with a SMF, they could synergistically enhance both the secretion and bioactivity of exosomes, for example, by enriching miR-1260a to promote angiogenesis and osteogenesis. Consistent with previous reviews highlighting the versatility of nanoparticles in bone tissue engineering [32], our study demonstrates that Fe_3_O_4_ magnetic nanoparticles, combined with physical stimulation, can serve not only as direct osteoinductive agents but also as effective preconditioning tools for enhancing exosome function. While mineral-based nanoparticles have been extensively explored for their intrinsic osteoinductive properties when incorporated into scaffolds or hydrogels, our approach leverages them in a novel context: as cellular primers to generate therapeutically enhanced exosomes. This strategy differs fundamentally from studies utilizing bone-derived nanoparticles (BNPs), which directly deliver extracellular matrix components to MSCs to modulate Notch signaling and promote differentiation [33,34]. Instead, our work establishes that Fe_3_O_4_ and SMF preconditioning reprogram BMSCs to produce exosomes with dual antioxidant and pro-osteogenic functions, operating via the miR-429/NOG pathway. This cell-free approach may offer advantages in terms of scalability and consistency compared to direct cell or BNP-based therapies, representing a distinct mechanistic avenue for bone regeneration.

On a mechanistic level, small RNA sequencing analysis revealed the upregulation of multiple miRNAs in BMSC-Fe_3_O_4_-SMF-Exos. A literature survey indicates that among these, rno-miR-339-3p has been reported as a tumor suppressor in cancer [53], rno-miR-500-3p can promote tumor progression [54], and rno-let-7i-3 has been shown to inhibit tumor growth [55]. Most relevant to the bone metabolism and oxidative stress regulation focus of this study is rno-miR-429. Previous research suggests its role in alleviating oxidative stress, protecting osteoblasts, and promoting osteogenesis. Therefore, we focused on miR-429 and, for the first time, investigated it within the context of repairing radiation-induced bone injury. This study further clarifies that exosome-delivered miR-429 directly targets and inhibits NOG, a key antagonist of the BMP signaling pathway, thereby promoting osteogenic differentiation. Notably, while previous literature has suggested the antioxidant potential of miR-429, our work identifies a novel mechanism through which it regulates osteogenesis via NOG in the context of radiation-induced bone damage. Our findings suggest that miR-429 may synergistically break the “oxidative stress-impaired osteogenesis” vicious cycle by exerting a dual function: mitigating oxidative stress directly or indirectly, and concurrently relieving the suppression of the pro-osteogenic BMP/Smad pathway through NOG inhibition, thereby collectively fostering bone repair. However, whether the antioxidant and pro-osteogenic effects are causally linked or simply act in parallel remains to be elucidated by further investigation involving precise modulation of ROS levels in conjunction with analyses of this pathway.

This study also has several limitations. First, the mechanistic investigation was primarily conducted in vitro. The in vivo microenvironment of radiation-induced bone injury is extremely complex, involving interactions among vascular, immune, and nervous systems, which may introduce additional influencing factors. Whether the miR-429/NOG axis remains the dominant mechanism in animal models requires direct validation through in vivo experiments. Second, exosomal cargo is highly complex and heterogeneous [56]. Beyond miR-429, other differentially expressed miRNAs, proteins, or lipids identified by sequencing may exert synergistic or additive effects. Future studies should employ in vivo rescue experiments using specific miR-429 inhibitors or Nog overexpression for further confirmation. Third, the long-term biosafety, in vivo biodistribution, and potential immunogenicity of exosomes pre-conditioned with Fe_3_O_4_ NPs necessitate systematic evaluation before clinical translation.

To address these limitations and strengthen the translational relevance of our findings, future studies should employ established preclinical models of radiation-induced bone injury. The mandibular osteoradionecrosis (ORN) model in rats or rabbits, involving a single high-dose radiation (15–20 Gy) to the mandible followed by tooth extraction, closely mimics clinical pathology [57,58]. Alternatively, the rat femoral or tibial irradiation model can be used to assess bone regeneration in long bones [57]. Critical-sized calvarial or mandibular bone defects in irradiated animals would allow direct evaluation of exosome-mediated bone regeneration [59,60]. Outcome assessments should include micro-CT analysis for bone volume and microarchitecture, histomorphometry for new bone formation, immunohistochemistry for osteogenic markers (RUNX2, OCN) and vascularization (CD31), as well as tracking of fluorescently labeled exosomes to confirm their homing to injured bone tissue. Furthermore, mechanism-validation experiments using Noggin-overexpressing transgenic animals or local delivery of miR-429 antagomirs could confirm the in vivo relevance of the miR-429/NOG axis. Such studies would not only confirm our proposed mechanism but also establish the translational feasibility of BMSC-Fe_3_O_4_-SMF-Exos as a cell-free therapy for radiation-induced bone injury.

## 5. Conclusions

In summary, this study successfully developed a novel exosome-based strategy for the therapy of radiation-induced bone injury. By preconditioning BMSCs with a combination of Fe_3_O_4_ MNPs and a static magnetic field, we generated exosomes (BMSC-Fe_3_O_4_-SMF-Exos) with dual-enhanced “pro-osteogenic” and “antioxidant” functions. These exosomes effectively rescued the osteogenic differentiation capacity of irradiated BMSCs and alleviated oxidative stress. Mechanistically, their therapeutic effect was mediated via exosomal delivery of miR-429, which targeted and inhibited NOG, thereby activating the BMP/Smad signaling pathway. This work not only provides a promising cell-free therapeutic strategy for bone regeneration following radiation injury but also offers new insights into the interplay between oxidative stress mitigation and bone repair.

## Data Availability

The original contributions presented in this study are included in the article/Supplementary Material. Further inquiries can be directed to the corresponding authors.

## Supplementary Materials

The following supporting information can be downloaded at: https://www.mdpi.com/article/10.3390/bioengineering13040402/s1, Figure S1. Colloidal stability characterization of the synthesized Fe_3_O_4_ MNPs. Figure S2. Particle size distribution of Fe_3_O_4_ MNPs analyzed from TEM images. Table S1. Sequences of qRT-PCR primers used in this study.

## Abbreviations

The following abbreviations are used in this manuscript:

MNPs	Magnetic nanoparticles
SMF	Static magnetic field
BMSC	Bone marrow mesenchymal stem cells
Exos	Exosomes
NOG	Noggin
ROS	Reactive oxygen species
BMP	Bone morphogenetic protein
ALP	Alkaline phosphatase
RUNX2	Runt-related transcription factor 2
OCN	Osteocalcin
COL1A1	Collagen type I alpha 1 chain
MDA	Malondialdehyde
SOD	Superoxide dismutase
RNS	Reactive nitrogen species
PI3K/Akt	Phosphatidylinositol 3-kinase/protein kinase B
MAPK	Mitogen-activated protein kinase
HDAC7	Histone deacetylase 7
COL4A2	Collagen type IV alpha 2 chain
SD	Sprague-Dawley
MEM	α Minimum Essential Medium alpha
FBS	Fetal bovine serum
EDTA	Ethylenediaminetetraacetic acid
PEG	Polyethylene glycol
TEM	Transmission electron microscopy
SEM	Scanning electron microscopy
EDS	Energy-dispersive X-ray spectroscopy
XPS	X-ray photoelectron spectroscopy
XRD	X-ray diffraction
NdFeB	Neodymium-iron-boron
PBS	Phosphate-buffered saline
NTA	Nanoparticle tracking analysis
qRT-PCR	quantitative real-time polymerase chain reaction
ARS	Alizarin Red S
DAPI	4′,6-Diamidino-2-phenylindole
GAPDH	Glyceraldehyde-3-phosphate dehydrogenase
U6	U6 small nuclear RNA
RIPA	Radio Immunoprecipitation Assay
BCA	Bicinchoninic acid
DCFH-DA	2′,7′-dichlorodihydrofluorescein diacetate
DHE	Dihydroethidium
WT	Wild-type
3′-UTR	3′untranslated region
MUT	Mutant
NC	Negative control
MFI	Mean fluorescence intensity
IR	Irradiated
Ms	Saturation magnetization
Hc	Coercivity
Mr	Remanence
