# Supplementary material for: Fe3O4 Magnetic Nanoparticles and Static Magnetic Field Stimulated BMSC-Derived Exosomes Promoted Osteogenesis and Alleviated Oxidative Stress in Irradiated BMSCs Through miR-429/NOG Pathway
Source: Bioengineering (Basel). 2026 Mar 30;13(4):402. doi: 10.3390/bioengineering13040402 (PMC13113114; doi:10.3390/bioengineering13040402)
Supplement: Supplementary file 1 [file bioengineering-13-00402-s001.zip › bioengineering-4187243-supplementary.pdf]

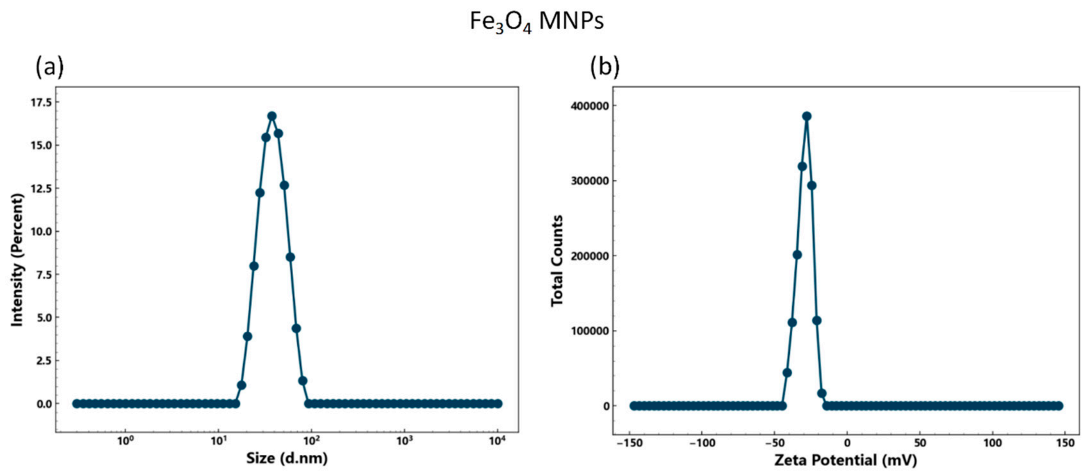

Figure S1. Colloidal stability characterization of the synthesized Fe_3O_4 MNPs.
(a) Hydrodynamic size distribution of Fe_3O_4 MNPs in ultrapure water (pH 7.4) measured by dynamic light scattering (DLS); **(b)** Zeta potential distribution of Fe_3O_4 MNPs in ultrapure water (pH 7.4).

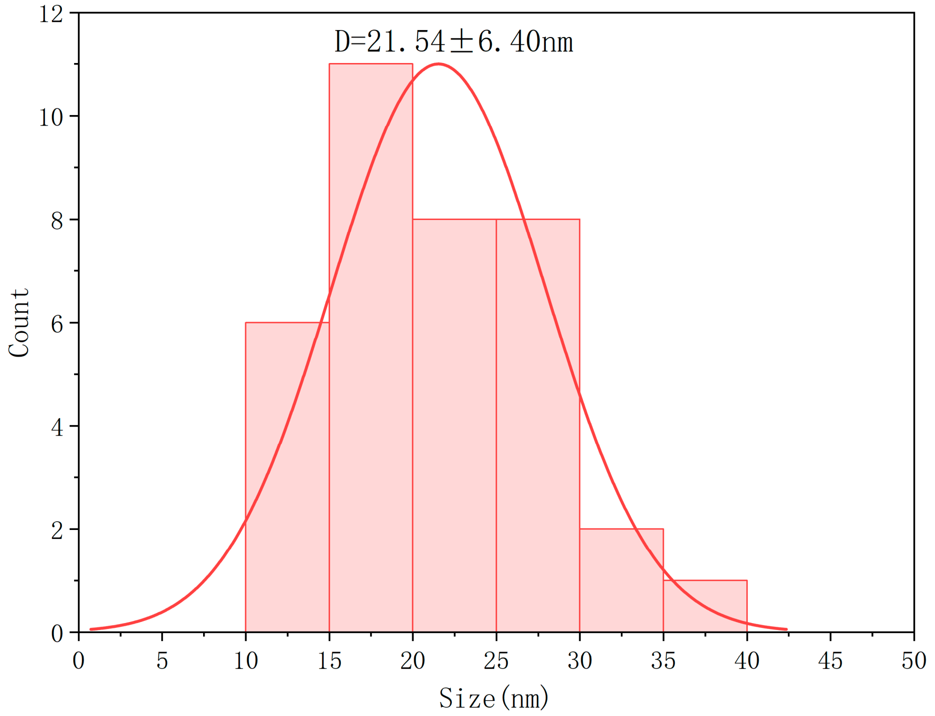

Figure S2. Particle size distribution of Fe_3O_4 MNPs analyzed from TEM images.
Histogram of particle size distribution (n=36) fitted with a Gaussian function, showing an average diameter of 21.54 ± 6.40 nm.
